# Mobile gene clusters and coexpressed plant–rhizobium pathways drive partner quality variation in symbiosis

**DOI:** 10.1073/pnas.2411831122

**Published:** 2025-07-29

**Authors:** Muhammad Rizwan Riaz, Ivan Sosa Marquez, Hanna Lindgren, Garrett Levin, Rebecca Doyle, Mario Cerón Romero, Julia C. Paoli, Jenny Drnevich, Christopher J. Fields, Barney A. Geddes, Amy Marshall-Colón, Katy D. Heath

**Affiliations:** ^a^Department of Plant Biology, University of Illinois Urbana-Champaign, Urbana, IL 61801; ^b^Department of Microbiology, University of Illinois Urbana-Champaign, Urbana, IL 61801; ^c^Carl R. Woese Institute for Genomic Biology, University of Illinois Urbana-Champaign, Urbana, IL 61801; ^d^Department of Microbiological Sciences, North Dakota State University, Fargo, ND 58102; ^e^Department of Biology, McMaster University, Hamilton, ON L8S 4K1, Canada; ^f^University of Illinois Urbana-Champaign, Roy J. Carver Biotechnology Center, Urbana, IL 61801

**Keywords:** symbiosis, systems genetics, dual-RNAseq, horizontal gene transfer, partner quality

## Abstract

Plant–microbe symbioses such as the legume–rhizobium mutualism are vital in the web of ecological relationships within both natural and managed ecosystems, influencing primary productivity, crop yield, and ecosystem services. The outcome of these interactions for plant hosts varies quantitatively and can range from highly beneficial to even detrimental depending on natural genetic variation in microbial symbionts. Here, we take a systems genetics approach, harnessing the genetic diversity present in wild rhizobial populations to predict genes and molecular pathways crucial in determining partner quality, i.e., the benefits of symbiosis for legume hosts. We combine traits, dual-RNAseq of both partners from active nodules, pangenomics/pantranscriptomics, and Weighted Gene Co-expression Network Analysis (WGCNA) for a panel of 20 *Sinorhizobium meliloti* strains that vary in symbiotic partner quality. We find that genetic variation in the nodule transcriptome predicts host plant biomass, and WGCNA reveals networks of genes in plants and rhizobia that are coexpressed and associated with high-quality symbiosis. Presence–absence variation of gene clusters on the symbiosis plasmid (pSymA), validated in planta, is associated with high or low-quality symbiosis and is found within important coexpression modules. Functionally our results point to management of oxidative stress, amino acid and carbohydrate transport, and NCR peptide signaling mechanisms in driving symbiotic outcomes. Our integrative approach highlights the complex genetic architecture of microbial partner quality and raises hypotheses about the genetic mechanisms and evolutionary dynamics of symbiosis.

## Significance

Addressing challenges from sustainable agriculture to ecosystem resilience is only possible by integrating symbiotic microorganisms into our biological understanding. The effects of symbionts on hosts percolate up from genomes through expression and metabolism to result in complex traits including nitrogen fixation and plant health. Here, we leverage a model nitrogen-fixing symbiosis, integrating genomes, traits, and gene expression to discover novel genes, gene clusters, and associated metabolic and regulatory processes that are important for the variable effects of bacteria on plant growth. We provide functional validation and insight into variation in symbiotic functioning and foundational information on the inheritance of important traits in microbial symbiosis. This raises exciting questions about how these keystone symbionts evolve in nature.

Plant–microbe symbioses form the foundation of a complex network of ecological interactions that drive primary productivity in both natural and managed ecosystems. These interactions can have positive, negative, or little impact on one or both species involved (1, 2) with mutualistic symbioses resulting in fitness benefits to both partners (3). Despite these categorical distinctions, the fitness benefits to partners in any mutualistic interaction vary quantitatively (4, 5) along a continuum depending on the environmental context (6–8) and the genotype of one or both partners (5, 9–15). Because the fitness-determining traits in symbiosis are often microscopic or biochemical, a predictive understanding of symbiosis in ecology, evolution, and agriculture requires a mechanistic approach that is grounded in functional genetics but leverages natural genetic variation (14).

Quantitative genetic trait variation, the focus of targeted breeding programs and selection in natural populations (16), is underlain by SNP variants, presence–absence variation (PAV) of genes and gene regions, and dynamic gene expression (17, 18). Despite the dominance of the single-locus central dogma viewpoint in bacterial genetics to date, this quantitative framework is salient for bacterial traits for several reasons. In contrast to sexual eukaryotes, for which genotype–phenotype mapping studies were developed (19, 20), bacterial recombination and genetic variation are not driven by reproduction but occur horizontally (21) as a result of both homologous and nonhomologous mechanisms of recombination, often involving mobile genetic elements (MGEs) (22). As a result, bacterial genomes are often highly dynamic and require reference-quality genome assemblies for optimum variant calling, MGE detection, and annotation of the full pangenome (all genes in a population) (23–27). What’s more, many symbiotic bacteria have multipartite genomes, which consist of two or more large replicons [i.e., chromosome, megaplasmids, or chromids (28, 29)], which can have vastly different demographic processes, genetic variation, recombination, and rates of PAV (5, 30–33). The expression of these multipartite genomes is highly plastic, with bacteria leveraging different genes or even whole replicons depending on the environment (34–36). Thus, a thorough understanding of ecologically and agriculturally relevant symbiosis requires that we quantify how pangenomic variation percolates up through gene expression networks and metabolic pathways to generate phenotypic variation.

Like all eukaryotes, plants require nitrogen (N) in accessible forms (nitrates and ammonia), which is often limiting in the soil, restricting growth. Leguminous plants overcome this limitation by forming symbiosis with a polyphyletic group of bacteria known as rhizobia, which inhabit root nodules and therein convert atmospheric N (*N_2_*) into plant-usable forms (3, 37–39). The legume–rhizobium symbiosis begins with symbiont recognition and infection, leading to nodule formation (40, 41), differentiation of rhizobia into bacteroids (37, 42), and finally resource exchange between partners (35, 43). The resulting mutualism is a key contributor to the nonanthropogenic N cycle, controlling N availability for legume crops, nonlegume crops through rotation, and primary producers in natural terrestrial ecosystems (44, 45).

The interaction between *Medicago truncatula* and *Sinorhizobium meliloti* is a critical model for studying symbiotic nitrogen fixation. Decades of work on this model legume–rhizobium symbiosis have revealed much about the genetics and biochemistry governing the interaction (46–49), and the effects of both host and symbiont genetic variation present in natural populations on host growth and fitness, sometimes called partner quality (5, 50, 51). Functional genetic and –omics studies have culminated in hundreds of genes known to be required for symbiosis (52, 53) and an increasingly detailed understanding of symbiotic metabolism (54–57). Natural populations harbor abundant variation in symbiotic compatibility and efficiency maintained via largely unknown genetic and evolutionary processes occurring in recognition and signaling genes, metabolic pathways, and other molecular mechanisms (36, 38, 58). Our understanding of these genetic underpinnings of partner quality variation in nature remains nascent, though this knowledge holds promise for manipulating symbiotic relationships to enhance agricultural productivity and for predicting how environmental changes will impact nitrogen cycling. Thus, in addition to molecular genetic approaches that typically focus on one or a handful of well-characterized genotypes, studying the genetic variation in the symbiotic transcriptome across genotypes from natural populations grown in a controlled, common garden design can provide a handle with which to resolve novel pathways involved in high-quality symbiosis, while also highlighting which known pathways are variable and thus likely to be a source of evolutionary innovation in natural populations.

Here, we take a systems genetics approach (59–62) leveraging standing genetic variation present in wild populations of rhizobia, transcriptomics using dual-RNAseq of host and symbiont gene expression from active symbiotic nodules (34), and pangenomes for a panel of 20 wild rhizobial isolates known to vary in partner quality (5), in order to predict the underlying molecular pathways/genes most related to partner quality in nitrogen-fixing symbiosis. We show how plant and symbiont gene expression networks interact with the presence–absence of functional gene clusters to drive mutualistic partner quality variation.

## Results

### Variation in the Nodule Transcriptome and Presence–Absence of Gene Clusters on pSymA Predict Host Plant Biomass.

To examine the molecular mechanisms by which rhizobial genetic diversity influences partner quality with its host plant, we inoculated *M. truncatula* cv. DZA 315.16 (hereafter DZA) plants with 20 individual rhizobia strains (*SI Appendix*, Fig. S1) from natural populations of *S. meliloti;* these strains were previously shown to induce a diverse range of phenotypes on this host (5). In the present study, we measured 10 phenotypes from host plants (Dataset S1) (10 biological replicates per *S. meliloti* strain). A trait correlation network of the phenotypes showed that plant shoot biomass was positively correlated with root biomass, chlorophyll, leaf number, and height, and negatively correlated with nodule number (*SI Appendix*, Fig. S2). Plant shoot biomass was highly variable across strains (σ^2^ = 0.024; CV = 0.60; *P* < 0.0001; *SI Appendix*, Fig. S3). Hereafter, we refer to strains that confer high to low biomass accumulation as high-, intermediate-, and low-quality partners, respectively (48, 50).

**Fig. 1.**
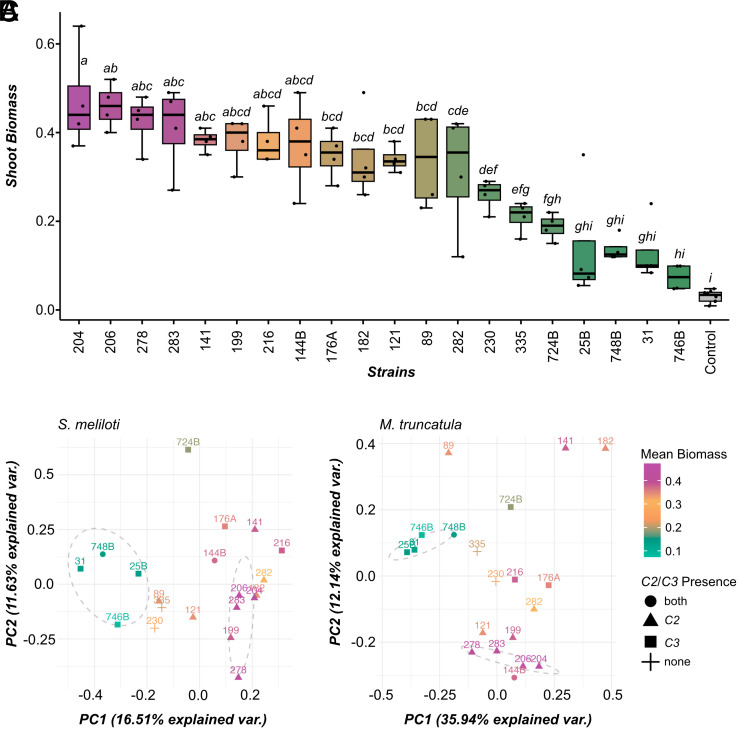
Partner quality of 20 *S. meliloti* strains and PCA of nodule transcriptomes. (*A*) Boxplots present the distribution of plant biomass when *M. truncatula* host plants were inoculated with one of 20 strains (four replicates each) or remained uninoculated (control). Letter-based groupings of means were assigned by ANOVA followed by Duncan’s test (alpha = 0.05). (*B*) PCA plot derived from the expression of 5,993 *S. meliloti* genes (mapped to *S. meliloti* 1021 reference), (*C*) PCA plot derived from the expression of the 5,000 most highly variable genes of *M. truncatula*. The transcriptome profiles are quantified using average *logCPM* values of expressed genes. Each data point on the PCA plot corresponds to a strain; data points closer together are more similar in terms of gene expression patterns than those further away. Each data point is color-coded according to the mean biomass value of the respective strain. Data point shape represents the presence or absence of distinct gene clusters (C2, C3) in strains.

We obtained symbiotic transcriptomes by extracting nodule RNA from a subset of four biological replicates per strain for RNA-seq analysis (Illumina paired-end reads ranging from fifty-eight to hundred million reads). We first mapped transcripts to *M. truncatula* [A17 *r5.0* downloaded from the French National Research Institute for Agriculture, Food and Environment Medicago Bioinformatics Resources (63)] and *S. meliloti* (reference strain 1021 assembly GCF_000006965.1 ASM696v1) (64, 65). Approximately 44 to 87% of total reads were mapped with this reference-based analysis (includes coding and noncoding genes), where 12.4 to 96.3% of those reads mapped to *Medicago,* depending on the sample (Dataset S2). To uncover how natural genetic variation across rhizobium strains influences the expressed genome, we first used principal component analysis (PCA) to find that partner quality variation (same replicates as RNAseq; Fig. 1*A*) was, overall, associated with transcriptomic variation. Importantly, PCA on strain means revealed that *S. meliloti PC1* explains 16.5% of the variability in the transcriptome and separates strains by partner quality (Fig. 1*B*). Similarly, Medicago PC1 and PC2 together explain 48.1% of transcriptome variability and separate strains by partner quality (Fig. 1*C*).

Most annotated genes (77.7% of the *S. meliloti* 1021 genome) are commonly expressed (counts ≥ 10 in all replicates) across strains, while 3% of genes are not expressed in any strain (counts < 10 in all replicates). Examination of the remaining genes (19.3%) revealed on/off patterns of expression where certain genes are expressed in some strains and not expressed in others (*SI Appendix*, Fig. S4). The binary nature of these genes affords us the opportunity to perform a discrete analysis of functional expression variation across strains. Thus, we imposed a filtering criterion to obtain a refined list of genes that are only expressed in at least three but not more than 17 strains, resulting in 574 genes with on/off expression patterns. Of these, 82 genes significantly correlate with shoot biomass (|r | > 0.5, fdr < 0.15).

To determine whether the on/off expression patterns were caused by PAV in the bacterial genomes, whereby the lack of expression would be the result of not having the gene, we generated high-quality de novo assemblies of all 20 strains using PacBio long-read sequencing. *BLAST+* sequence search of these closed, reference-quality genomes confirmed that 54 of the 82 genes with on/off expression patterns are also PAVs based on the 1021 reference (Dataset S3; see *Materials and Methods* for gene selection criteria). Next, we performed a pangenome analysis to establish the full set of core and variable gene content (in our 20 strains, plus the 1021 reference strain and the newly released reference strain MABNR56 assembly GCF_037023865.1 ASM3702386v1). The fraction of the core (present in all strains), shell (variable presence across strains), and cloud (rare) gene groups varied among the main genome elements; pSymA contained a much smaller fraction (19.9%) of core genes compared to either the chromosome (56.1%) or pSymB (67.9%; *SI Appendix*, Fig. S5). In addition, eight strains feature a smaller, fourth accessory plasmid (BioProject PRJNA1009820). We found 60 additional open reading frames (ORFs) that were present at intermediate frequency across the 20 wild strains, absent in the 1021 reference, and significantly correlated with shoot biomass (|r| > 0.5, fdr < 0.15).

**Fig. 2.**
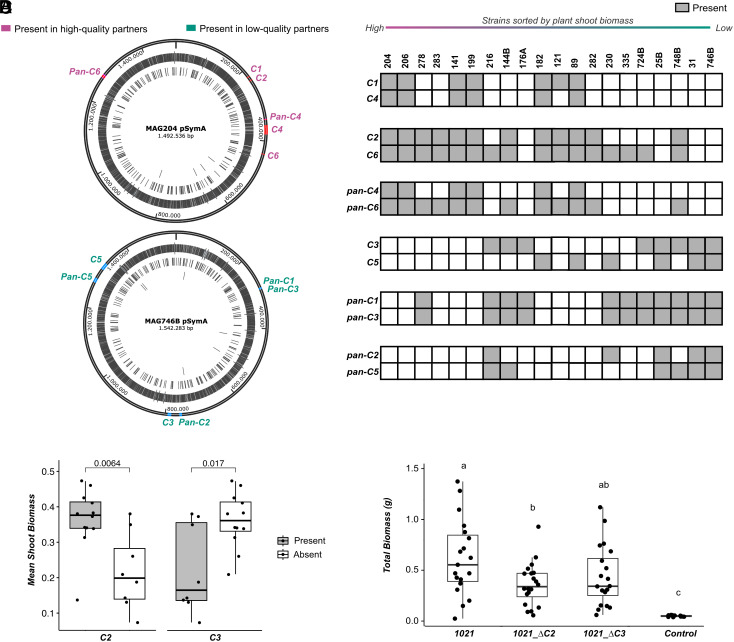
Gene cluster PAV associated with partner quality. (*A*) Circular map of the symbiotic plasmid (pSymA) of MAG204 and MAG746B showing the location of 1021-based (*C1-C6*) and pangenome-based (*panC1-panC6*) gene clusters. (*B*) Matrix displaying the presence or absence of gene clusters. Rows represent cluster names (*C1-C6, panC1-panC6*), and columns represent strains, sorted from high to low shoot biomass. Gray boxes indicate the presence of the cluster in the strain. (*C*) Mean shoot biomass of *M. truncatula* in symbiosis with strains in which clusters C2 and C3 are either present (gray) or absent (white). Each data point represents the mean shoot biomass of a strain (20 strains from the current experiment), and group means are compared through an independent *t* test. Significance levels are shown for each comparison. (*D*) Total biomass of *M. truncatula* inoculated with 1021, deletion mutants of clusters C2 (1021_ΔC2) and C3 (1021_ΔC2), or mock inoculated (control). Significantly different means are based on Tukey post hoc test.

The 54 “reference-based” and additional 60 “pangenome” PAV loci of interest were largely found on pSymA, versus pSymB or the chromosome. Fifty-one “reference-based” pSymA genes are clustered into six syntenic groups, henceforth called clusters C1 (2 genes), C2 (4 genes), C3 (10 genes), C4 (14 genes), C5 (17 genes), and C6 (4 genes) (Fig. 2*A* and Dataset S3)—plus one single IS3-type transposase. An additional two transposases that form part of these 54 genes are located in pSymB (IS630-like element) and the chromosome (IS256-like element). Thirty-three “pangenome” PAV loci were also clustered into six groups (defined as two or more ORFs within 3 kb of each other) on pSymA, henceforth called clusters pan-C1 (2 ORFs), pan-C2 (8 ORFs), pan-C3 (3 ORFs), pan-C4 (2 ORFs), pan-C5 (11 ORFs), pan-C6 (7 ORFs) (Fig. 2*A* and Dataset S3). Clusters C1, C2, C4, C6, pan-C4, and pan-C6 are present in high-quality strains, while clusters C3, C5, pan-C1, pan-C2, pan-C3, and pan-C5 are mostly present in low-quality strains (Fig. 2*B* and Dataset S3). Strains that show intermediate phenotypes have different combinations of high- and low-quality associated gene clusters (strains in the center of Fig. 2*B*).

### Reference-Based Clusters for Validation and Functional Analysis.

Focusing on the clusters found using the 1021 annotation, since this strain can be readily manipulated (53, 66), the presence/absence patterns of C2 and C3 were most associated with host DZA shoot biomass across the 20 *S. meliloti* strains (Fig. 2*C*). We confirmed the relevance of these gene clusters in partner quality variation by deleting clusters C2 (1021-ΔC2) and C3 (1021-ΔC3) in reference strain 1021. Consistent with our prediction based on our 20 strains, *Medicago* cv. DZA inoculated with 1021-ΔC2 had a 42% decrease in plant biomass compared to DZA inoculated with 1021 (*P* = 0.024; Fig. 2*D*). While we expected loss of Cluster 3 to increase DZA plant biomass, given its association with low-quality strains in this natural population, we found that plants inoculated with 1021-ΔC3 were not statistically different from those inoculated with 1021 (*P* = 0.124; Fig. 2*D*).

Cluster C2 (286,919 to 288,703 in ref. *S. meliloti* 1021) has functions related to N export, flavonoid signal transduction, and nodulation and includes an *Lrp/AsnC* family transcriptional regulator (*WP_234826226.1*). This TF family has been shown in *Escherichia coli* and *Bacillus subtilis* to have global and/or specific regulatory functions; in *E. coli,* AsnC is known to regulate asparagine synthetase (*asnB*). Asparagine has been reported as an important form of N produced in nodules and used by host legumes, and thus could be critical for host benefits. Cluster C2 also contains two SnoaL-like domain-containing proteins (*WP_010967276.1*/UniProt: Q930B7, *WP_010967277.1*) of the *NTF2* superfamily (IPR032710) that can reportedly interact with proteins binding to flavonoid molecules involved in the communication between a close relative to *S. meliloti, Rhizobium etli,* and its host plant. There is also a class I SAM-dependent methyltransferase protein (*WP_010967274.1*/ UniProt: B3KLX3/GO:0008757) that is related to NodS (SAM) proteins involved in oligosaccharide biosynthetic processes, although the specific function of this gene in *Medicago-Sinorhizobium* symbiosis is unknown.

### PAV on the pSymA Phylogeny Indicates Mobility of Gene Clusters.

Heterogeneity in the PAV of gene clusters within well-defined clades of the core pSymA phylogeny (composed of the superset of 191 strains; *SI Appendix*, Fig. S6*A*) indicates the gain and loss of clusters through time, suggesting mobilization of these genetic elements via unidentified mechanisms. For example, a closer inspection of the pattern of both PAV and copy number of Cluster C2, even within our 20-strain subset, suggests cluster duplication and gene gain/loss through the evolutionary history of pSymA. First, the presence of C2 is scattered across the core strain phylogeny (strains in bold in *SI Appendix*, Fig. S6*B*). Second, four strains have two copies, suggesting recent acquisition of a second cluster. Third, drilling down even further by creating a phylogeny of the Cluster C2 itself (using an alignment of C2 gene sequences from strains where the cluster is present; *SI Appendix*, Fig. S6*C*) reveals two things: that the topology of the C2 phylogeny is distinct from the pSymA core genome topology, and that the C2 copies found in the same strains are not recent duplicates – again indicating independent movement of C2 relative to pSymA.

### The Nodule Pantranscriptome Reveals Coexpression Modules That Correlate with Gene Clusters on pSymA and Partner Quality.

Next, we explored pantranscriptomic variation by realigning our RNAseq data to the *S. meliloti* pangenome and *M. truncatula* reference and employed coexpression analyses to study how variation in bacterial gene expression is related to patterns of PAV in gene clusters and strain partner quality. Of all 12,107 gene groups identified in the pangenome, 39.6% were commonly expressed (counts ≥ 10 in all replicates), 4.7% were not expressed, and 55.7% were variably expressed across strains (Dataset S4). While ORFs in the core genome were more highly expressed than those in the accessory (shell and cloud) genome, there was higher dispersion (variance in gene expression among strains) in the accessory genome (*SI Appendix*, Fig. S7).

**Fig. 3.**
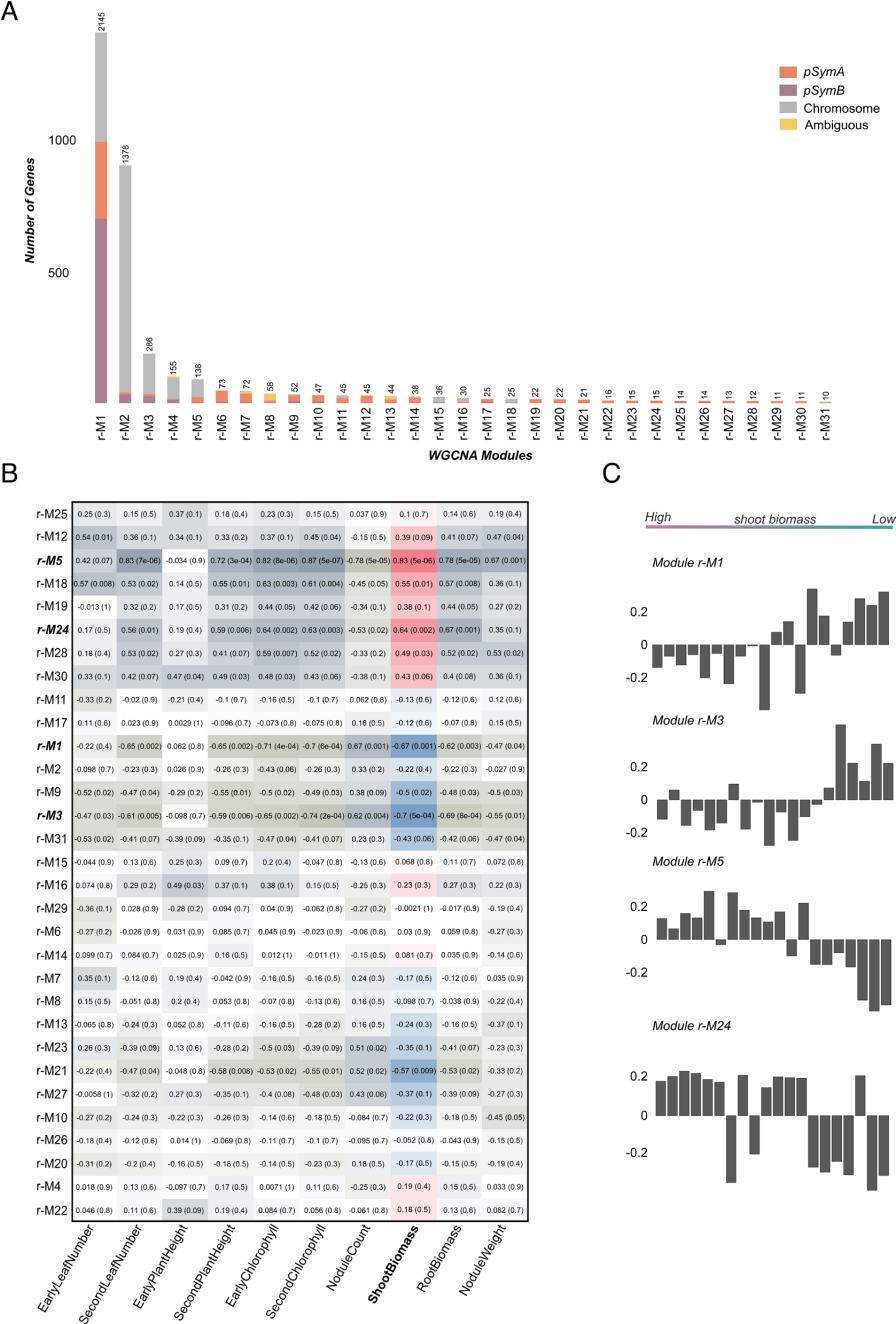
WGCNA of *S. meliloti* transcriptome. (*A*) Barplot showing the number of genes per WGCNA module, ordered by module size. The colors indicate the proportion of chromosomal and plasmid genes in each module. (*B*) Heatmap displaying the correlation coefficient (and *P*-value) between module eigengenes (ME) (rows) and plant traits (columns). The modules are sorted based on their similarity to each other. Highly significant modules (|r| > 0.6) are bolded and italicized. Correlations between modules and host plant biomass are highlighted (pink: positive; blue: negative). (*C*) The bar plots display eigengene values for significantly correlated modules for each strain, sorted from high to low shoot biomass along the *X* axis. Expression of genes in modules M5 and M24 are positively correlated with shoot biomass, while those in M1 and M3 are negatively correlated.

Our weighted gene coexpression network analysis (WGCNA) grouped genes into 31 modules representing the genetic variation in the bacterial gene network (Fig. 3*A* and *SI Appendix*, Fig. S8) (67). Modules vary in size from 10 to 2,145 genes, and some are enriched for either the chromosome, pSymA, or pSymB (Fig. 3*A*). Next, we correlated these modules with partner quality traits. The largest modules were dominated by chromosomal and pSymB genes and tended to be negatively correlated with biomass (Fig. 3 *A* and *B*); of 31 modules, only two were significantly positively associated with shoot biomass, while two were negatively correlated with shoot biomass (|r| > 0.6, *P* < 0.05) (Fig. 3 *B* and *C*). Genes were labeled as ambiguous when they were found on different genomic elements (e.g., chromosome, pSymA, pSymB, or accessory plasmids) across the strains in our study. Interestingly, two modules (r-M8 and r-M13) feature many of these potentially mobile genes, though neither module was significantly associated with plant biomass (Fig. 3 *A* and *B*). Three pangenome PAV clusters, pan-C2, pan-C4, and pan-C5 were filtered out from WGCNA (median expression < 10 counts); however, while these three clusters were present in only 5 to 6 strains, they were expressed when present (Dataset S5).

The two modules positively correlated with host shoot biomass include r-M5 (|r| = 0.83, *P*-value 4e-06), and r-M24 (|r| = 0.64, *P*-value 0.002) (Fig. 3*B*). These modules were closely related in hierarchical clustering of WGCNA modules (*SI Appendix*, Fig. S8); r-M5 is enriched with chromosomal genes, and r-M24 is enriched with pSymA genes (Fig. 3*A* and Dataset S6). Notably, r-M24 includes many of the transcriptional regulation related PAV genes of clusters C2 and pan-C6, which are coexpressed with genes encoding the type II toxin–antitoxin (TA) system. This includes *RelB/E* family loci [*WP_010967242.1* (A), *WP_010967243.1* (T)] located on pSymA. Deletion of these two genes in a different symbiotic context (RmP110 with congeneric **Medicago* sativa*) was not found to significantly affect N fixation (68), though other TA systems are known to influence symbiosis (69, 70), and we found that knocking out C2 in Sm1021 did decrease plant biomass. Apart from TA systems, r-M24 also includes Clustersof Orthologous Groups (COG) terms for transcriptional regulation such as *Lrp/AsnC* (*WP_014532001.1*) and arabinose regulatory mechanisms (*GlxA; WP_014989851.1*, and *AraF; WP_010975369.1*), and an SRPBCC-domain containing protein (*WP_127535573*), an effector protein shown to promote symbiosis (71). However, no COG terms were significantly enriched in r-M24. Module r-M5 is enriched in COG terms for energy production and conversion (fdr < 0.0001) and coenzyme transport and metabolism (fdr < 0.0001), and includes many important N fixation genes on pSymA including *fixABCGIKOUX* and *nifABDEHKNX* (*SI Appendix*, Fig. S9 and Datasets S6 and S7) (53, 72, 73).

The two modules negatively correlated with host shoot biomass include r-M1 (|r| = 0.67, *P*-value 0.001), and r-M3 (|r| = 0.7, *P*-value 0.0005) (Fig. 3*B* and Dataset S6). Module r-M1 features mostly (~80%) pSymB and chromosomal genes (Fig. 3*A*) and is enriched (fdr < 0.0001) in 13 COG categories including carbohydrate transport (by ABC transporters) and metabolism, transcription, and lipid transport and metabolism (Dataset S7). Module r-M3 includes mostly genes on the chromosome (Fig. 3*A*) and is enriched (fdr < 0.0001) in posttranslational modification, turnover, chaperones and transcription (Dataset S7). Although they were not found in the top modules, clusters pan-C1 and pan-C3 were both found in r-M21, which was marginally negatively correlated with shoot biomass (r = −0.57; Dataset S6).

### Plant-Bacteria Coexpression Reveals Host and Bacterial Modules That Are Correlated with Each Other and with Plant Biomass.

**Fig. 4.**
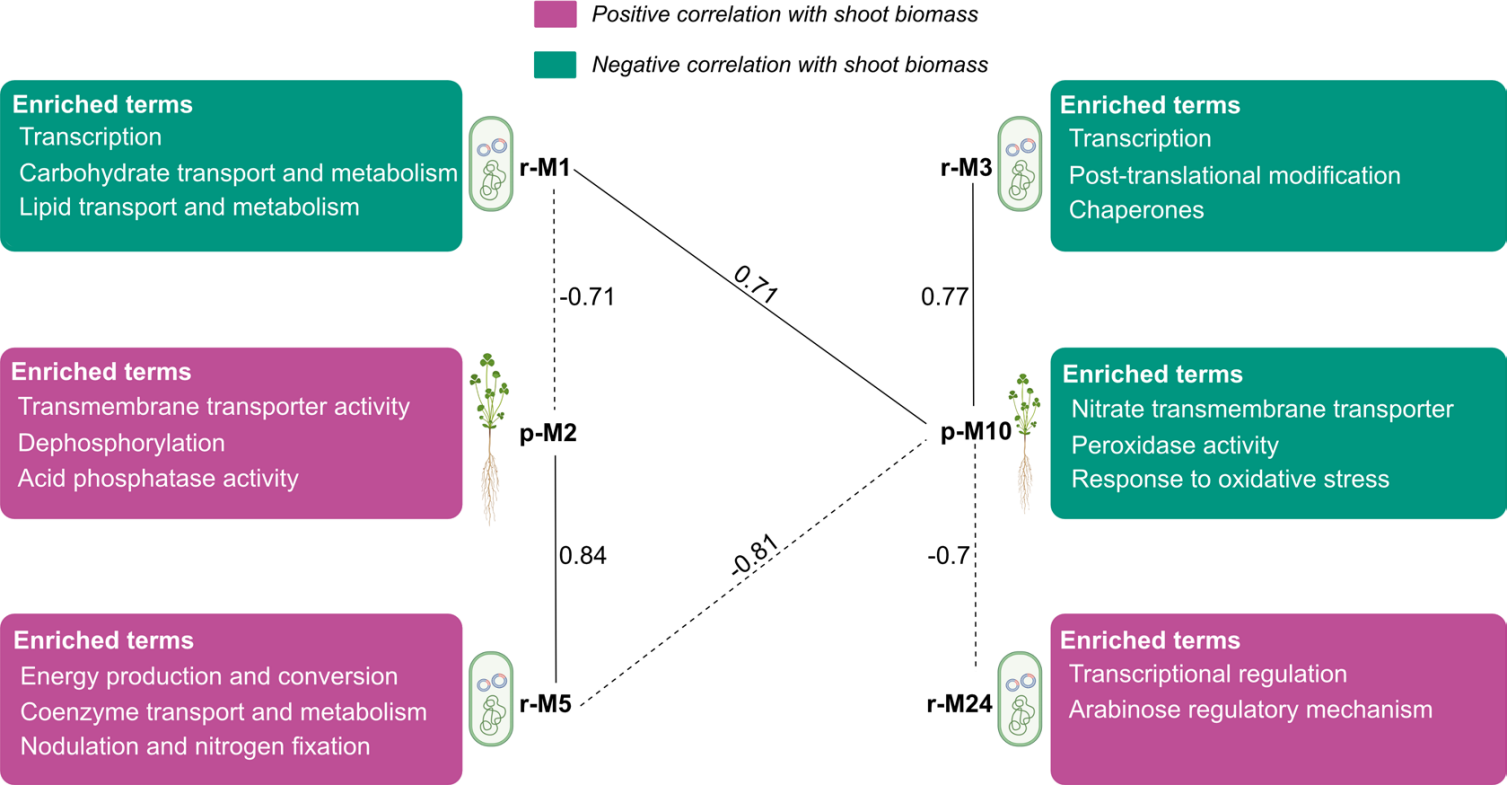
Relationships among important coexpressed plant and rhizobia modules. Pearson correlations between MEs of plant and rhizobia modules are shown. Enriched COG/GO terms are shown for each module. Negative correlations are represented as dotted lines, positive correlations as solid lines.

We also studied how plant transcriptomes varied across partner rhizobium strains and identified plant gene modules (Dataset S8) significantly correlated with shoot biomass (more details in the *SI Appendix, Supplemental Text*). To examine the coexpression of plant and rhizobium genes within the nodule, we correlated modules across both networks (plant and rhizobia) using Module Eigengenes (MEs). Two plant (p) modules (p-M2 and p-M10) were significantly correlated with both rhizobium modules (cutoff: |*r*| > 0.7) and with plant shoot biomass (|*r*| > 0.6) (Fig. 4 and Dataset S9). We investigated these and closely related modules to uncover the relationships among legume and rhizobial biological processes regulating biomass accumulation.

The plant module p-M2 and its close relatives p-M5 and p-M14 are positively correlated with shoot biomass and negatively correlated with r-M1 and r-M3 (Fig. 4 and Dataset S9). The representative Gene Ontology (GO) terms for the p-M2 module are release and recycling of phosphorus, the formation and function of nitrogenase via PPIases and Fe binding, and systemic resistance (genes listed in Dataset S10). Module p-M5 is enriched in GO terms related to protein and messenger RNA (mRNA) turnover, while p-M14 genes are enriched in GO terms for the synthesis of protein and mRNA (Dataset S10); together, these terms suggest transcriptional and translational reprogramming toward the benefit of plant biomass accumulation.

The second plant module p-M10, along with close relatives p-M4 and p-M35, are negatively correlated with shoot biomass and are positively correlated with rhizobia modules r-M1 and rM3 while negatively correlated with r-M5 and r-M24 (Fig. 4 and Dataset S9). Representative GO terms for module p-M10 include oxidative stress and nitrate transmembrane transport (Fig. 4 and Dataset S10); module p-M4 is enriched in GO terms for O2 binding and transport, killing of cells of another organism, and mRNA processing. The p-M4 module is dominated by late nodulin genes, a category of nodule-specific cysteine-rich (NCR) peptides that are frequently expressed in ineffective nodules (Dataset S8) (74), these late nodulin genes are also expressed in module p-M35 that is enriched in GO terms related to the import of proteins into the inner membrane of the mitochondria.

Putting it all together, we find that cluster PAV underlies expression patterns between plant and bacterial gene modules associated with partner quality. Specifically, in higher-quality strains (containing the transcriptional regulators in C2 and pan-C6), the expression of genes in modules r-M5–r-M24–p-M2 (Fig. 4) is coordinated such that genes involved in defense against toxins and oxidative stress (r-M24) and canonical nitrogen-fixation (*nif/fix*) genes (r-M5) are coexpressed with plant genes (p-M2) involved in phosphorus turnover (needed to enhance N-fixation and nodule respiration), ferric and ferrous iron binding, and protein peptidyl-prolyl isomerization, all necessary components for the proper folding and function of nitrogenase in effective nodules (75–79). Moreover, in these high-quality strains, the coordinated down-regulation of bacterial genes involved in carbohydrate and lipid transport and metabolism, along with plant genes encoding NCR peptides and oxidative stress responses, are associated with increased biomass accumulation. As expected, opposite expression patterns are observed in lower-quality strains (containing C3 and not C2) that result in less biomass accumulation. These interrelationships – gene cluster PAV, coordinated host-bacterial coexpression, and plant benefits—emphasize the complexity of the genetic and regulatory underpinnings of natural variation in N-fixing symbiosis.

## Discussion

Managing legume–rhizobium symbiosis for sustainability and agricultural improvement and understanding symbiosis evolution in natural populations both require a systems-level analysis grounded in standing genetic variation (62). Recent strides in genomics, transcriptomics, and computational biology, along with abundant natural diversity within the *M. truncatula*-*S. meliloti* symbiosis have created opportunities for large-scale analyses of active processes within established nodules (34, 49, 80). These processes drive the trade of resources that ultimately determines the degree to which plants benefit from rhizobial symbiosis (54, 63, 81–83). Leveraging the naturally occurring genetic diversity across 20 *S. meliloti* strains, we employ a systems genetics approach that combines dual-RNAseq transcriptomics of host and symbiont with long-read, reference-quality genomes and phenotypic data to 1) highlight bacterial genes and pathways that contribute to partner quality variation available to natural and artificial selection for symbiotic improvement, 2) discover reveal variation in pSymA gene presence–absence that contributes to plant benefits, and 3) resolve coordinated host-symbiont coexpression in the nodule pantranscriptome associated with plant benefits.

### Genetic Architecture and Inheritance of Rhizobium Partner Quality.

Our research supports growing evidence that partner quality variation in nature depends on complex genetic architecture, including the modular inheritance of genes on symbiotic plasmids (84–87), particularly pSymA in *S. meliloti*. Nucleotide variations in the core genes of *S. meliloti* and their response to the environment (G x E) have previously been linked with quantitative natural variation in host benefits, as highlighted in the recent Genome-wide association study (GWAS) study by Batstone et al. (2022) and others (88). Here, we refine our understanding of the genetic architecture of partner quality in *S. meliloti* by adding the presence/absence of pSymA gene clusters, as well as correlated gene expression changes in thousands of genes in both host and symbiont. The association between PAV in functionally relevant gene clusters and partner quality in our dataset, along with incongruent evolutionary histories of clusters compared to the pSymA phylogeny, suggest that horizontal mobility of symbiosis genes, and their modularity in linked gene clusters, are likely major forces that generate variation in partner quality (36, 67, 85). This is particularly noticeable with cluster C2, which was found in high-quality strains in our population and was even found in duplicate in a subset of our strains including (but not limited to) some of the highest-quality strains (204, 199, 141). Whether copy number or simply presence of these putative symbiosis gene clusters is more important for symbiotic function remains to be determined; both are known to be important in *Mesorhizobium* (21). Knocking out C2 in the laboratory reference strain 1021 caused a major reduction in plant biomass, validating the association between C2 and partner quality. Integrating across diverse studies that draw from quantitative and population genetic approaches (33, 89), mapping (5, 21, 51), experimental evolution (4, 90–92), and transcriptomics (9, 49, 80, 93) suggests a model of symbiotic partner quality in which hundreds of diverse genetic underpinnings, and thus diverse modes of transmission and patterns of selection, determine the benefits of symbiosis for hosts in nature.

Our experimental design, in which a single genotype of hosts was inoculated with one of 20 strains in a common garden, allowed us to quantify how genetic variation in *Sinorhizobium* contributes to expression variation in both plant and rhizobium genes. Here, changes in plant gene expression are plastic responses of the plant transcriptome to genetic variation residing in the bacterial genome (i.e., symbiotic extended phenotypes; 4). Variation in *Sinorhizobium* gene expression, on the other hand, could be due to variants within the expressed genes themselves or variants elsewhere in the rhizobium genome and might involve multigene interaction (44, 66), epigenetic modifications (94, 95), or even structural variants/rearrangements that influence expression but not gene content (96). Remarkably, 56 out of 60 “universal” candidate genes (those mapped in at least three experiments) from our previous GWAS using a much larger set of strains (5) were actively expressed in nodules, suggesting that natural variation at the loci themselves, as well as expression of these loci, play a role in partner quality. We found 54 of these genes in modules significantly negatively correlated with shoot biomass (43 genes in r-M1 and 11 genes in r-M10; Dataset S11). Together these approaches provide robust targets for novel gene discovery and spotlight the role of natural variation in amino acid, polyamine, and sugar transporters in optimal symbiosis (*WP_010967350.1;SM_RS26550,WP_010967351.1;SM_RS26555,WP_010975345.1;SM_RS19440*; Dataset S11). They also highlight the importance of misregulation and miscommunication in poor-quality symbiosis, since a regulator and solute transport were associated with smaller shoot biomass (*WP_010975343.1;SM_RS19430, WP_010975557.1;SM_RS20515*; Dataset S11). Though these genes are novel, these functional hypotheses fit with our current understanding of bacterial metabolism, which is largely based on studies of a single laboratory strain (1021) and its isogenic mutants (29, 66, 97). Additional functional studies would be necessary to resolve the molecular mechanisms by which variation at these particular loci influence symbiosis, but these are tantalizing candidates given that transport functions typically lack resolution in metabolic models (54–57).

One implication of our findings is that clusters of genes associated with decreased partner quality in our study appear to be horizontally transmitted and maintained at intermediate frequency in natural *S. meliloti* populations. There are several hypotheses that might explain these results. First, selection during other phases of the bacterial lifestyle, beyond the nodules, might increase the frequency of these clusters if they improve bacterial fitness in other environments. For example, Burghardt et al. (2018) found little relationship between the relative fitness of strains in symbiosis versus soil, indicating environment-dependent selection on rhizobia. Second, it is possible that these same clusters are beneficial in other host genotypes; indeed, the partner quality of different *Sinorhizobium* strains is well known to depend on the host genotype (4, 5, 93, 98), implying that the phenotypic effect of alleles in those strains interact with genes in the host [i.e., intergenomic epistasis (98)]. Similarly, the effects of clusters on host and symbiont fitness might depend on epistatic interactions with other genes in the rhizobium genome; indeed, while our initial analysis indicated that cluster C3 was associated with low-quality partners, knocking out this cluster in the reference led to nonsignificant effects on partner quality—suggesting epistasis. Finally, fitness in multiscale symbiosis (including, for example, plasmids within bacteria and bacteria within hosts) (99) might play a role in maintaining seemingly unfit genes (86). There is a growing recognition that horizontally transmissible elements including plasmids, transposons, and other types of MGEs [are known to be abundant on pSymA (100)] have their own fitness interests and agency in natural populations (67). If gene clusters that decrease partner quality actually increase the frequency with which the MGE that carries them is duplicated, transmitted, or permitted entry, they might persist even in the face of plant-mediated selection for high quality rhizobia (67, 99, 101).

### Cross-Membrane Transport Systems and Signaling Pathway Heterogeneity Are Major Drivers in Plant Cell and Bacteroid Communication and Mutualistic Coordination.

Our dual-RNAseq of both plant and rhizobium genes actively expressed in nodule tissue enables us to identify functional processes in both partners that are strongly correlated with each other and with plant biomass. Modules positively associated with plant biomass (r-M5, r-M24, and p-M2) were mutually enriched in oxidative protection and energy mechanisms for the synthesis of N fixation genes along with the necessary plant processes and cofactors needed for the formation, stabilization, and function of the nitrogenase complex. Examining processes negatively correlated with plant biomass (r-M1, r-M3, and p-M10, p-M4, p-M35) suggests a role for plant NCR signaling genes expressed in low-quality interactions (102, 103) (Dataset S8). NCRs are activated in successive waves during nodule organogenesis and exhibit specific spatial localization of their transcripts from the apical to the proximal nodule zones (104, 105). Diverse NCR molecules have been associated with both infection response mechanisms and positive signaling for mutualism in compatible interactions, but also the terminal differentiation of bacteroids and ongoing maintenance of mutualism (102, 103, 106, 107). Given that we studied the genetic variation in gene expression across rhizobium strains, plant NCR expression is plastically responding (directly or indirectly) to genetic factors in the genomes of low-quality strains (104, 108). The roles of bacterial effectors, NCRs, and their coordinated bacterial responses are still being worked out using functional genetic approaches (109); nevertheless, it is known that NCRs can result in arrested development of bacteroids in symbiosomes (110, 111) and that strains have different susceptibilities to NCRs depending on both the presence of particular loci [e.g., BacA-like proteins (105)] as well as the genetic variation in unknown genes (110, 111). Importantly, all of our strains improve plant growth over control (Fig. 1*A*); thus, it is possible that NCR overexpression in our study signals plant response to nonterminally differentiated bacteroids due to incompatibilities with strains, rather than a defense response to rhizobia that would decrease plant fitness. It is also important to note that some NCRs in our study and in others promote high quality symbiosis (15).

It is important to note that there are ~200 annotated *ABC transporters* in *S. meliloti*, significantly more genes than in other similar alpha-proteobacteria (81, 112); nevertheless, our dataset is enriched in these functions compared to expectation based on their frequency in the genome. ABC transporters are heterogenic transmembrane protein systems that consist of the adenosine triphosphate-binding cassette-transporter, whose function is to actively mobilize solutes across the lipid membrane of the cell (113). Our study raises questions about communication dynamics between both partners in symbiosis, particularly involving ABC transporters and membrane permeability systems. Molecule transportation systems are thought to be determinants of mutualistic symbiosis between rhizobia and legumes and other organisms such as arbuscular mycorrhizae (114) and are particularly important for the communication between bacteroid and plant cells in the symbiosome as a means of nutrient mobilization and metabolic integration (97). The positive association of certain bacterial modules (e.g., r-M5 and r-M24) that contain various transporters (i.e. lipid, protein, AA, carbohydrate, etc.) with plant modules (p-M2) for membrane functions and amino acid metabolism (sulfur, sugar, nitrogen, metal ion, etc), but negative association with plant nitrate transmembrane transport (p-M10), suggests microbial membrane transport influences gene regulation in the plant cell and ultimately plant growth.

### Conclusions.

Systems genetics approaches have the potential to resolve the genetic mechanisms underlying ecologically and economically important variation in complex traits such as partner quality in nitrogen-fixing symbiosis. The contribution of host–strain interactions, G x E interactions, epistasis, and modular gene clusters suggest complex genetic architecture and Horizontal gene transfer underlying partner quality variation, shedding light on the fundamental nature of trait variation and inheritance in microbial populations. Identifying novel loci involved in oxidative stress, respiration, cross-membrane transport, carbon and nitrogen metabolism, and particular plant NCRs for future genetic studies will add resolution to mechanistic models of symbiosis, while providing a model for how host-symbiont coexpression analysis can generate hypotheses about coordinated functions in symbiosis. Our results also raise new evolutionary questions about how functional gene cluster PAV is maintained despite longstanding and abundant evidence for fitness alignment (via plant partner choice and/or sanctions) that increases the fitness of high-quality rhizobia (115–120). Our integrated approach thus provides valuable insights into the genomic and molecular intricacies of symbiotic interactions but also sets the stage for future research directions in understanding the evolution and sustainability of legume–rhizobium partnerships for agricultural improvement.

## Materials and Methods

### Experiment Design and Plant Measurements.

We grew host plants, *M. truncatula* (DZA), in a factorial, single inoculation experiment with 20 *S. meliloti* strains; 10 plants per strain were phenotyped (200 plants, plus controls), and four replicates were used for nodule RNA extraction (80 plants total). Plants and strains were chosen to maximize partner quality variation based on previous observations and were grown in the greenhouse as previously described (5). We took data on leaf number and height early in the experiment (~2 and 4 wk after planting). During harvest (6 wk postplanting), we measured several additional indicators of rhizobium partner quality: shoot and root dry biomass, chlorophyll content using a SPAD 502DL plus Chlorophyll meter (Spectrum technologies, inc. Bridgend, UK) and total number of nodules. For RNA, mature nodules (47 per plant on average) were collected from *Medicago* roots (mean nodule weight = 77 mg) into steel-bead prefilled tubes, immediately flash-frozen in liquid nitrogen, and stored at −80 °C. Frozen nodules were homogenized using a prechilled bead beater machine and stored back at −80 °C after total tissue disruption. We used the Plant RNeasy kit from QIAGEN® (MD, USA), adding an additional bead beater step while tissue was in lysis buffer, to obtain an average of 348 ng/μL (45.3-1282.1) of pure RNA.

### Sequencing, Data Preprocessing, and Reference-Based Transcriptome.

RNAseq libraries were prepared with Illumina’s TruSeq Stranded mRNAseq Sample Prep kit (Illumina, CA, USA), then pooled, quantitated by qPCR, and sequenced on one S4 lane for 300 cycles from both ends of the fragment on a NovaSeq 6000 sequencing system (Illumina). Fastq files were generated, demultiplexed with the bcl2fastq v2.20 Conversion Software (Illumina), and adaptors were trimmed. *M. truncatula* (A17 *r5.0*) reference files downloaded from INRAE/CNRS Medicago Bioinformatics Resources (63), and *S. meliloti* (strain 1021 assembly GCF_000006965.1 ASM696v1) reference files were downloaded from NCBI (64, 65). Salmon (121) (v 1.4.0) was used to index the combined *M. truncatula* (mrna + ncrna + rrna files) and *S. meliloti* (cds + rna files) transcriptomes using the decoy-aware method with the entire *M. truncatula* + *S. meliloti* genomes as the decoy sequence. Then quasi-mapping was performed to map reads to the combined transcriptomes with additional arguments --*seqBias, --gcBias, --numBootstraps*=30, --*validateMappings*, and --*recoverOrphans* to help improve the accuracy of mappings. Sequence data can be found in the Gene Expression Omnibus under accession number GSE212235. Additional statistical analyses were done in R (122) (v4.1.0) using packages as indicated in *SI Appendix*.

### Identification of 1021-Based PAV in *S. meliloti*.

The PAV of genes in each strain was determined by establishing counts per million (CPM) cutoffs for each strain. Genes with expression below the cutoff in three out of four replicates are labeled as “absent” in that strain, following a criterion adapted from the *filterByExpr* function in the *edgeR* package (123, 124) in R (122) (v4.1.0). Further filtering based on gene presence–absence patterns resulted in a set of 574 genes (Dataset S11) that were present in at least three and a maximum of 17 strains; of those, genes highly correlated with plant shoot biomass were selected based on cutoff (|r| > 0.5). PAV of these candidates were further verified with the *BLAST+* (125) search of gene sequences (cutoff: at least 98% identity and query coverage to call presence) against long-read assemblies of 20 strains (BioProject PRJNA1009820). We kept only candidate genes that had a 100% agreement between the predicted presence/absence calls based on the expression pattern and the sequence search results. Additional details can be found in the *SI Appendix*.

### Long-Read Assemblies of 20 *S. meliloti* Strains and Pangenome Assembly.

DNA extraction was performed using the PacBio Nanobind CBB kit® (PacBio, USA) on cultivated bacterial cells obtained from 20 strains of *S. meliloti*, then DNA extracts processed for PacBio Sequel II HiFi sequencing (126). Consensus long-read assemblies for the 20 *S. meliloti* strains were constructed using the Trycycler® tool (127), using default parameters, setting a minimum of 100× reads. To create neighbor-joining trees of the core genomes of the *S. meliloti* chromosome, pSymA, and pSymB, we aligned genomes and created trees using the SPINE-NUCMER-SNPS pipeline (128), then edited trees in FigTree (v1.4.4) and ggtree in R (v4.1.0).

Core, Shell, and Cloud categories across the pangenome were based on their presence across strains. Genes were grouped by their genomic element (chromosome, plasmid pSymB, plasmid pSymA, or eight nondefined extra chromosomal elements). Genes were then classified as “Core” (>99% of strains), “Shell” (15 to 95% of strains), or “Cloud” (<15% of strains) (129). The data were summarized by counts of gene groups and proportions calculated relative to the total of annotated genes for each element. Genome assemblies for all *S. meliloti* samples, including the 20 strains plus two reference genomes (Strain 1021 assembly GCF_000006965.1 ASM696v1; MABNR56 assembly GCF_037023865.1 ASM3702386v1), along with relevant annotation, were processed using Panaroo v1.5.1 (130) to generate a pangenome network for all protein-coding genes from the 22 *S. meliloti* strains. Full methods can be found in the *SI Appendix*.

### Pantranscriptome Gene Expression Quantification and Summary Statistics.

Salmon v1.10.0 (121) was used to generate a combined *M. truncatula* + *S. meliloti* pantranscriptome reference database. First, we combined MtrunA17r5.0 mRNA, ncRNA, and rRNA transcript FASTA files [downloaded from INRAE/CNRS Medicago Bioinformatics Resources (63)] with the *S. meliloti* pangenome “combined_DNA_CDS.fasta” transcripts, along with decoys consisting of the full MtrunA17r5.0 genome FASTA and the two NCBI *S. meliloti* reference genomes, ASM696v1 and ASM3702386v1. Salmon was then used to quantify the counts per transcript. Depending on the sample, the percentage of reads aligned to the combined *Medicago + S. meliloti* pangenome was 3.1 to 35.9%, of which 20.8 to 95.7% mapped to *Medicago* (Dataset S2). While this total alignment percentage appears much lower than the reference-based transcriptome, it does not include the ~7 to 75% rRNA per sample, only protein-coding transcripts. Full methods can be found in the *SI Appendix*.

### Genetic Manipulation of 1021-Based Gene Clusters C2 and C3.

Gene cluster deletions in *S. meliloti* Rmp110 were generated via an Flippase-Flippase Recognition Target recombination system: Plasmids with flanking FRT sites were constructed using Golden Gate cloning (131), introduced into *E. coli*, and then mobilized into *S. meliloti* by triparental mating. Next we performed a common garden experiment in the greenhouse to assess the effect of modified strains on *M. truncatula*. Plants were inoculated with mock control, wild-type strain 1021, or deletion mutants (1021_ΔC2, 1021_ΔC3), then grown for 8 wk. Aboveground tissue was harvested for biomass measurements, and we used one-way ANOVA and Tukey post hoc tests of variation among inoculation treatment. Full methods including a full list of bacterial strains and plasmids can be found in the *SI Appendix*.

### WGCNA.

We used the strain means of gene expression (average *logCPM*) to construct the coexpression networks in the WGCNA (132) package in *R* (122) (v4.1.0). The networks were constructed separately for *M. truncatula* and *S. meliloti* using the Medicago reference genome and pangenome of *S. meliloti* along with two reference strains.

Kyoto Encyclopedia of Genes and Genomes (KEGG) Automatic Annotation Server (133) was used for assigning KEGG Orthology numbers (Sinorhizobium and Medicago) and mapping of genes to metabolic processes and BRITE categories. The COG annotation of reference *Sinorhizobium* genes was obtained from *eggNOG-mapper* (134). KEGG and COG categories assignments were used to evaluate the enrichment of gene sets using *ClusterProfiler* (135) package in R (122) (v4.1.0). Full methods can be found in the *SI Appendix*.

## Supplementary Material

Appendix 01 (PDF)

Dataset S01 (XLSX)

Dataset S02 (XLSX)

Dataset S03 (XLSX)

Dataset S04 (XLSX)

Dataset S05 (CSV)

Dataset S06 (CSV)

Dataset S07 (CSV)

Dataset S08 (CSV)

Dataset S09 (TXT)

Dataset S10 (CSV)

Dataset S11 (CSV)

Dataset S12 (XLSX)

Dataset S13 (CSV)

## Data Availability

R scripts of the analyses are available on GitHub (https://github.com/mrizwanriaz/symbiosis) (136). Long-read assemblies can be accessed at NCBI BioProject PRJNA1009820 (137). Bulk RNAseq raw reads of *M. truncatula* and *S. meliloti* are available on GEO accession GSE212235 (138).
