# Supplementary material for: Mobile gene clusters and coexpressed plant–rhizobium pathways drive partner quality variation in symbiosis
Source: Proc Natl Acad Sci U S A. 2025 Jul 29;122(31):e2411831122. doi: 10.1073/pnas.2411831122 (PMC12337268; doi:10.1073/pnas.2411831122)
Supplement: Supplementary file 1 — Appendix 01 (PDF) [file pnas.2411831122.sapp.pdf]

PNAS

Supporting Information for

Mobile gene clusters and co-expressed plant-rhizobium pathways drive partner quality variation in symbiosis

Muhammad Rizwan Riaz[1+], Ivan Sosa-Marquez[1+], Hanna Lindgren[1], Garrett Levin[4], Rebecca Doyle[1,2], Mario Cerón Romero[1], Julia C. Paoli[1], Jenny Drnevich[3], Christopher J. Fields[3], Barney A. Geddes[4], Amy Marshall-Colón[1,*], Katy D. Heath[1,*]

[1]University of Illinois Urbana-Champaign, Department of Plant Biology, Urbana, IL, USA; [2]McMaster University, Hamilton, ON, CA;

[3]University of Illinois Urbana-Champaign, HPCBio, Roy J Carver Biotechnology Center, Urbana, IL, USA; [4]North Dakota State University, Fargo, ND

[+*]These authors contributed equally

*Amy Marshall-Colón and Katy Heath
Email: amymc@illinois.edu and kheath@illinois.edu

This PDF file includes:

Supporting Text
Tables S1-S7
Figures S1 to S11
Legends for Supplemental Files S1 to S13
SI References

Other supporting materials for this manuscript include the following:

Supplemental Files S1 to S13

Supplemental Results

Gene clusters associated with biomass represent diverse functional pathways

Although we did not validate the functional importance of the *C3* cluster in Sm1021 (529,340 – 539,369 in ref. *S. meliloti* 1021; present in low-quality partners), this cluster is located between essential symbiotic islands (*Nif, Nod* and *fix*) in 1021 (1) and contains functions annotated in transport, opine synthesis, and conjugation. It includes four ABC opine transporters in the *att* operon (File S3) that are reportedly involved in the transport of mannopine (2). These proteins are similar (~70%) to *A. tumefaciens* transporters reportedly involved in transporting mannopine, used in host colonization and infection as a nutrient source by *Agrobacterium* (3-5). Rhizopine (rhizobial opines) have been reported to have a role in bacterial survival in symbiosis (6). Furthermore, *C3* includes a *GntR* family transcriptional regulator (*rctR*) that plays an important role in the signal transduction cascade repressing plasmid conjugation, putatively by controlling the expression of the *rctCBA* operon (7-11). The importance of conjugation repression or activation in the active nodule is unclear, but evidence in *Bradyrhizobium* suggests an important role of conjugation-related genes in the symbiotic interaction with the host (12). Other *C3* cluster genes synthesize putative proteins like a class III aminotransferase (WP_010967499.1) and an SDR family oxidoreductase (WP_010967501.1) with an unclear function in symbiosomes.

Cluster C4 primarily contains genes involved in molecular transport through ABC transporters, regulatory functions (transcriptional regulators *TetR/AcrR* and *MarR*) and cell wall modification-related enzymes (transglycosylase, phospholipase), while C5 is focused on transport and metabolism, particularly amino acids and polyamines. C6 is composed entirely of hypothetical proteins with unknown functions.

The distribution of functional annotations across the six pan-clusters shows specialized roles in cellular processes. Specifically, *pan-C1* is mainly associated with transcriptional regulation, modulating gene expression via DNA binding and control mechanisms, given the involvement of the *Rrf2* family transcriptional regulator (WP_018098784.1) (13). Both clusters *pan-C2* and *pan-C6* are primarily linked to signal transduction, facilitating cellular responses through phosphorylation (WP_015242250.1) mediated by response regulators (WP_015242251.1) and electron transport mechanisms involving c-type cytochromes (WP_015242248.1) (14). Moreover, *pan-C3* and *pan-C5* show enrichment in metabolic

functions, performing activities that are critical for energy production and balance and gene regulation, exemplified by NAD(P)/FAD-dependent oxidoreductases systems (WP_020487185.1) (15). Lastly, *pan-C4* appears to serve both regulatory and metabolic roles, as suggested by the presence of the MFS transporter (WP_014531244.1) (16).

Modules of plant gene expression responds to rhizobium strain variation

Like the rhizobium pantranscriptome analysis, we used WGCNA to identify modules of plant genes with variable expression across strains and correlated with shoot biomass (Figure S10). We identified 52 modules ranging in size from 35 – 7,167 genes. Important modules were selected based on the correlation cutoff ($|r| > 0.7$, $p < 0.05$) between module eigengene (ME) and plant shoot biomass. Modules significantly (p-value < 0.0001) positively correlated with shoot biomass include p-M2 ($r = 0.94$), p-M8 ($r = 0.71$), p-M18 ($r = 0.72$), p-M20 ($r = 0.71$), and p-M23 ($r = 0.76$) and are enriched for GO terms (p < 0.05) including transmembrane transporter activity, dephosphorylation, acid phosphatase activity, auxin-activated signaling pathway, auxin biosynthetic process and cytokinin-activated signaling pathway. On the other hand, strong negative correlations with shoot biomass were found for modules p-M10 ($r = -0.93$), p-M35 ($r = -0.7$), and p-M41 ($r = -0.72$) (Figure S10). These modules are significantly (p < 0.05) enriched for nitrate transmembrane transporter activity, response to oxidative stress, mitochondrial import inner membrane translocase complex, carbohydrate transmembrane transport and sugar transmembrane transporter activity GO terms.

Several known *M. truncatula* genes essential for effective nodulation and symbiotic nitrogen fixation process (SNF) (17) also show a variable expression from high to low quality of symbiotic partners (Figure S11). Some of the known SNF genes of *M. truncatula* are present in important WGCNA modules – eight genes in p-M2 (positively correlated with shoot biomass) including Too Much Love (TML)-related Kelch-repeat containing F-box protein, *MtTML2,* that regulates nodule numbers (18) and two genes in p-M35 (negatively correlated with shoot biomass) (File S13), including the DEMETER (DNA demethylase) and Metal Tolerance Protein (MtMTP2) genes.

Supplemental Methods

Sequencing, data preprocessing, and reference-based transcriptome

Total RNA was sent to the Roy J. Carver Biotechnology Center at the University of Illinois Urbana-Champaign for preparation, sequencing, and analysis. The RNAseq libraries were prepared with Illumina's TruSeq Stranded mRNAseq Sample Prep kit (Illumina, California, USA), then pooled, quantitated by qPCR, and sequenced on one S4 lane for 300 cycles from both ends of the fragment on a NovaSeq 6000 sequencing system (Illumina). Fastq files were generated, demultiplexed with the bcl2fastq v2.20 Conversion Software (Illumina), and adaptors were trimmed. *M. truncatula* (A17 *r5.0*) reference files were downloaded from INRAE/CNRS Medicago Bioinformatics Resources (19), and *S. meliloti* (strain 1021 assembly GCF_000006965.1 ASM696v1) reference files were downloaded from NCBI (20, 21). Salmon (22) (v 1.4.0) was used to index the combined *M. trunculata (*mrna + ncrna + rrna files) and *S. meliloti* (cds + rna files) transcriptomes using the decoy-aware method with the entire *M. trunculata* + *S. meliloti* genomes as the decoy sequence. Then quasi-mapping was performed to map reads to the combined transcriptomes with additional arguments --*seqBias, --gcBias, --numBootstraps*=30, --*validateMappings*, and --*recoverOrphans* to help improve the accuracy of mappings. Sequence data can be found in the Gene Expression Omnibus under accession number GSE212235.

The remaining statistical analyses were done in R (23) (v4.1.0) using packages as indicated below. Gene-level counts were estimated from transcript-level counts using the "bias corrected counts without an offset" method from the *tximport* package (v1.20.0). This method provides more accurate gene-level count estimates and keeps multi-mapped reads in the analysis compared to the traditional alignment-based method (24). The reads were split into *M. truncatula* and *S. meliloti* genes, and additionally filtered out if they did not have at least 10 counts per sample in at least 4 samples, and Trimmed Mean of M values (TMM) (25) normalized counts were used for each analysis.

Principal Component Analysis (PCA) was performed using the *prcomp* function from the *stats* package in R (23) (v4.1.0). The genes that passed low-filtering criteria were used to calculate the mean expression for each strain. The *filter_by_variance* function of the BioNero package (26) was used to extract highly variable genes of *M. truncatula*.

Identification of 1021-based presence-absence variation in *S. meliloti*

The presence-absence variation of genes in each strain was determined by establishing counts per million (CPM) cutoffs for each strain. The CPM cutoff for any strain is calculated by dividing

the number of replicates (i.e., four) by the median library size of replicates of the strain. The cutoff adapts to the library sizes of the replicates of the strain and filters out lowly expressed and noise genes. Genes with expression below the cutoff in three out of four replicates are labeled as "absent" in that strain, following a criterion adapted from the *filterByExpr* function in the *edgeR* package (27, 28) in R (23) (v4.1.0). The Upset plot was constructed to show the intersection among strains using the *UpSetR* (29) package in R (23) (v4.1.0). Further filtering based on gene presence-absence patterns resulted in a set of 574 genes (File S12) that were present in at least three and a maximum of 17 strains; of those, genes highly correlated with plant shoot biomass were selected based on cutoff ($|r| > 0.5$). Because gene expression reflects the alignment of reads against the reference genome (*S. meliloti* 1021), we further verified the presence-absence variation (PAV) of these candidates with the *BLAST+* (30) search of gene sequences (cutoff: at least 98% identity and query coverage to call presence) against the long-read assemblies of 20 strains (BioProject PRJNA1009820). We kept only candidate genes that had a 100% agreement between the predicted presence/absence calls based on the expression pattern and the sequence search results.

Long-read assemblies of 20 *S. meliloti* strains and pangenome assembly

DNA extraction was performed using the PacBio Nanobind CBB kit® (PacBio, USA) on bacterial cells obtained from 20 strains of *S. meliloti.* These strains were cultivated in liquid Tryptone Yeast media (TY) for 48 hours at 30 degrees Celsius on a shaker. Subsequently, DNA extracts were forwarded to the UIUC Roy J. Carver Biotechnology Center for PacBio Sequel II HiFi sequencing (31).

Consensus long-read assemblies for the 20 *S. meliloti* strains were constructed using the Trycycler® tool (32), using default parameters, setting a minimum of 100X reads. Trycycler® facilitates the generation of consensus sequences from multiple sequence alignments, reconciling alternative sequences from contigs to resolve circularization and distinguishing different replicons. In *S. meliloti*, these replicons typically represent a chromosome, a chromid (pSymB), a megaplasmid (pSymA), and an extra-chromosomal unidentified element in some strains (Contig 004). Sequencing also revealed that strains *282* and *283* have identical genomes; nevertheless, epigenetic modifications may contribute to the observed transcriptomic and biomass differences (Figure 1), thus we retained both strains for downstream analysis. To create neighbor-joining trees of the core genomes of the

chromosome, pSymA, and pSymB, we aligned genomes and created trees using the SPINE-NUCMER-SNPS pipeline (33), then edited trees in FigTree (v1.4.4) and ggtree in R (v4.1.0).

Core, Shell, and Cloud categories across the pangenome were based on their presence across strains. Genes were grouped by their genomic element (chromosome, plasmid pSymB, plasmid pSymA, or 8 non-defined extra chromosomal elements). Genes were then classified as "Core" (>99% of strains), "Shell" (15–95% of strains), or "Cloud" (<15% of strains). The data was summarized by counts of gene groups and proportions calculated relative to the total of annotated genes for each element. Genome assemblies for all *S. meliloti* samples, including the 20 strains plus two reference genomes (Strain 1021 assembly GCF_000006965.1 ASM696v1; MABNR56 assembly GCF_037023865.1 ASM3702386v1), along with relevant annotation, were processed using Panaroo v1.5.1 (34) via the following command: 'panaroo -i files.fofn -o Panaroo-run --clean-mode strict --remove-invalid-genes -a pan --threads 24' to generate a pangenome network for all protein-coding genes from the 22 *S. meliloti* strains.

Identification of gene presence-absence variation in the pangenome of 20 strains

The Panaroo pipeline (34) predicted presence-absence of each gene group based on genome assemblies. The Panaroo presence-absence output comprises of 11,985 gene groups. These genes were further filtered based on our previous criteria (see above), i.e., select genes that are present in at least three and maximum 17 strains. It resulted in a set of 2,411 genes, which are used to compute the correlation between presence-absence of gene groups and plant shoot biomass. We found 483 gene groups that are significantly correlated with plant shoot biomass ($|r| > 0.5$), which contain our previously identified 1021 based gene clusters as well. Then, 436 of these are single copy genes. We filtered these for low counts (when present) based on read alignment to pangenome, which resulted in 431, and after excluding previous candidates, 386 are new candidates from pangenome analysis.

We divided presence-absence candidate genes into two groups i.e., 1021 and non-1021 specific genes. 32 of these candidates are present in 1021 reference strain, out of which 23 are positively and 9 are negatively correlated with shoot biomass. 22 gene groups are present on pSymA and 10 are located on pSymB of 1021 reference strain. The non-1021 candidates, 354 out of 386, are comprised of 32 positively and 322 negatively correlated gene groups with shoot biomass. Exploring further the frequency of these gene groups, we found that 289 of these are only present in three or four strains, but we are interested in gene groups

with intermediate presence-absence variation (present in at least 5 and maximum of 12 strains). We selected 60 gene groups with intermediate variation, out of which 18 are positively correlated (meaning these genes are present in high-quality strains), and 42 are negatively correlated with shoot biomass.

We looked for strain-specific gene clusters in these candidates i.e., genes that are present consecutively in a strain. Next, we checked for clusters at pangenome level, and looked for gene groups present in clusters in all strains (when present in a strain), and identified six pangenome-level clusters comprising of 33 genes located on pSymA (File S3).

Pantranscriptome gene expression quantification and summary statistics

Salmon v1.10.0 (22) was used to generate a combined *Medicago truncatula* + *S. meliloti* pantranscriptome reference database. First, we combined MtrunA17r5.0 mRNA, ncRNA and rRNA transcript FASTA files (download from INRAE/CNRS Medicago Bioinformatics Resources (4)) with the *S. meliloti* pangenome "combined_DNA_CDS.fasta" transcripts, along with decoys consisting of the full MtrunA17r5.0 genome FASTA and the two NCBI *S. meliloti* reference genomes, ASM696v1 and ASM3702386v1. Salmon was then used to quantify the counts per transcript using the following options: 'salmon quant -i $REFERENCE -l ISF -1 $SAMPLE_ID_R1_001.fastq.gz -2 $SAMPLE_ID_R2_001.fastq.gz --numBootstraps=30 --validateMappings --recoverOrphans -o $SAMPLE_ID --seqBias --gcBias --writeUnmappedNames -p 12'.

The original combined 189,921 *Medicago* + *S. meliloti* pangenome transcripts were reduced to 95,428 unique transcript sequences by Salmon, which removes exact sequence duplicates when making the reference Salmon index database, because: 1) the same ORFs were duplicated due to their presence in multiple strains and thus multiple IDs in the full pangenome output, and 2) a small number of genes with multiple copies in strain genomes (e.g., transposases) are assigned separate gene group IDs and transcript IDs by Panaroo given their distinct genomic locations. Final transcripts pre- and post-duplicate filtering were: *Medicago* total = 50,343, unique = 49,928; *S. meliloti* total = 139,578, unique = 45,500. Panaroo's "gene_data.csv" output file was used to map *S. meliloti* protein-coding IDs to pantranscriptome gene cluster IDs, then sum all *S. meliloti* Salmon-based protein-coding gene counts (e.g., analogous to 'transcript-level' counts for *Medicago*) to gene cluster counts using

the “lengthScaledTPM” method from the tximport package (8) (v1.32.0) which additionally corrects for differences in protein-coding gene length.

Next, we used the presence-absence annotation for each gene group, along with expression counts, to calculate mean expression abundance and dispersion between core and accessory genes in the pantranscriptome (35). Genes not present in any of the 20 strains were filtered out, and remaining 11,454 gene groups were classified into “core” (4,926), if present in all 20 strains, and “accessory” (2,073), if present in 2-19 strains. The log_2CPM values were used to calculate the mean expression abundance and dispersion (mean absolute deviation), and two-sided Wilcoxon signed-rank test in R (23) (v4.2.2) was performed to compare core genes versus accessory genes (Figure S7).

Genetic manipulation of 1021-based gene clusters C2 and C3

The full list of bacterial strains and plasmids can be found in the tables below (Table S1-S7). *Escherichia coli strains* were routinely grown at 37°C on LB. *Sinorhizobium meliloti* strains were routinely grown at 30°C on LB supplemented with 2.5 mM $MgSO_4$ and 2.5 mM $CaCl_2$. When appropriate, the following concentrations of antibiotics were used when growing *E. coli*: chloramphenicol (Cm) 5 µg mL^{-1}, gentamicin (Gm) 20 µg mL^{-1}, kanamycin (Km) 25 µg mL^{-1}, and tetracycline (Tc) 5 µg mL^{-1}. The following concentrations of antibiotics were used when growing *S. meliloti*: gentamicin (Gm) 60 µg mL^{-1}, neomycin (Nm) 200 µg mL^{-1}, streptomycin (Sm) 200 µg mL^{-1}, and tetracycline (Tc) 5 µg mL^{-1}. Blue-white screening occurred on LB selection plates with a concentration of 40 µg mL^{-1} of 5-bromo-4-chloro-3-indolyl-beta-D-galactopyranoside (X-gal). 2.5 mM protocatechuate was included in mating plates to induce flippase production of pTH2505.

Deletions were created using an FLP-*FRT* site-specific recombination strategy (36). In brief, this approach relied on the ability of a flippase (FLP) to initiate the recombination of two *FRT* sites that flanked a desired region to be excised. Conjugation via triparental mating (37) was used to facilitate the transfer of either plasmids bearing a left or right flanking *FRT* site or a protocatechuate inducible flippase (pTH2505). Plasmids bearing left or right flanking *FRT* sites were constructed using Golden Gate cloning (38). All primers used to amplify homologous flanking regions are listed in the tables below. Putative plasmids were introduced to chemically competent DH5-Alpha cells via heat shock transformation. Along with selection

on antibiotics, blue-white screening via *lacZ* was utilized to identify recombinant bacteria. A complete list of assembled plasmids can be found in the table below.

Integration of plasmids bearing *FRT* sites into the genome of RmP110 (Rm1021 pSymA NC_003037) occurred via single-crossover homologous recombination. These integrations occurred step-wise, as the left flanking *FRT* site was first integrated, followed by the right flanking *FRT* site. Integration of these plasmids into the correct location of the RmP110 genome was verified using PCR. Primers and pairings used are listed in the tables below. After the presence of both the left and right *FRT* sites was verified, a final conjugation introduced a plasmid bearing a protocatechuate inducible flippase (pTH2505). Transconjugants were selected for on Sm and Tc and screened for Nm^R and Gm^S. Removal of the region flanked by *FRT* sites was verified by PCR. Primer pairings are listed below.

To evaluate the effects of modified *S. meliloti* strains on *M. truncatula*, we performed a fully-randomized common garden experiment in which DZA plants were inoculated with one of four treatments: mock inoculation, wild-type 1021, 1021_ΔC2, or 1021_ΔC3. After two weeks of germination, each plant was inoculated with 500 mL of bacterial culture adjusted to an optical density at 600 nm (OD600) of 0.1. Plants were grown for a total of eight weeks under controlled greenhouse conditions, at which time all plants were harvested and aboveground tissue weighed for biomass (our proxy for rhizobial partner quality). We used one-way ANOVA and Tukey post-hoc tests in R v4.1.0 to test for effects of gene deletions.

Pangenome-based Weighted Gene Co-expression Network Analysis (WGCNA)

We used the reads aligned to pangenome and Medicago reference to perform WGCNA. The strain means of gene expression (average *logCPM*) were used to construct the co-expression networks in the WGCNA (39) package in *R* (23). The networks were constructed separately for *M. truncatula* and *S. meliloti*. The genes with low/no expression are filtered out prior to performing the analysis. First, the network topology analysis was performed to determine the best soft-threshold power (β) using biweight midcorrelation and signed hybrid network in the *pickSoftThreshold* function. We computed the soft-threshold power for two subsets of data for performing WGCNA: Sinorhizobium pangenome genes (β = 10) and *Medicago* genes (β = 8) according to scale-free topology criteria. Subsequently, we used the WGCNA package to construct a weighted adjacency matrix that provides continuous connection strength ([0, 1]) using the selected soft-threshold power (Figure S8). Finally, the co-expression matrix and the

topological overlap matrix (TOM) were used to assess the interconnectedness of two genes by the degree of their shared neighbors across the global network. For each dataset (rhizobial genes and plant genes), the modules were detected computationally using *deepSplit* = 2 and *mergeCutHeight* = 0.2 parameters. The module eigengene (ME) is the first principal component of a given module. To find the most significant associations, MEs were used to compute the module membership (MM) and correlation with trait means.

Supplementary Tables related to transgenic methods

Table S1: Strain and Plasmid Table

Strain	Description	Reference
Sinorhizobium meliloti		
**RmP110**	Wildtype Derived from Rm1021 with Corrected *pstC* Allele	**Yuan et al. (40)**
**RmND868**	RmP110, Cluster 2 Deletion (RmP110 pSymA nt 286,591 – 288,859) via RmND861 with pTH2505 FLP; Sm^R Nm^R Tc^R	**This Work**
**RmND869**	RmP110, Cluster 3 Deletion (RmP110 pSymA nt 528,867 – 539,706) via RmND862 with pTH2505 FLP; Sm^R Nm^R Tc^R	**This Work**
**RmND861**	RmND842 with pNDMS494 Integrated; Sm^R Nm^R Gm^R	**This Work**
**RmND862**	RmND843 with pNDMS496 Integrated; Sm^R Nm^R Gm^R	**This Work**
**RmND842**	RmP110 with pNDMS493 Integrated; Sm^R Nm^R	**This Work**
**RmND843**	RmP110 with pNDMS495 Integrated; Sm^R Nm^R	**This Work**
Escherichia coli		
**DH5-Alpha**	*fhuA2Δ(argF-lacZ)U169 phoA glnV44 Φ80Δ(lacZ)M15 gyrA96 recA1 relA1 endA1 thi-1 hsdR17*	**New England BioLabs**
**MT616**	MM294A *recA-56* (pRK600), Mobilizer; Cm^R	**Finan et al. (37)**

**Plasmid**	**Description**	**Reference**
**pNDGG012**	Narrow Host Range Plasmid with *FRT* Site. L1-pMB1-Gm-*FRT*-ELT4; Golden Gate Assembly from pNDGG021, pOGG009, pNDGG007, pNDGG009, and pOGG014; Gm^R	**Geddes et al. (38)**
**pNDGG013**	Narrow Host Range Plasmid with *FRT* Site. L1-p15A-Km-*FRT*-ELT4; GG Assembly from pNDGG021, pOGG008, pNDGG006, pNDGG009, and pOGG014; Km^R	**Geddes et al. (38)**
**pTH2505**	*flp* Gene Controlled by Protocatechuate Inducible Promoter in pRK7813; Tc^R	**Zhang et al. (41)**
**pNDMS493**	pNDGG013 with RmP110 pSymA nt 286,239 – 286,590 (GLND512/GLND513) via BsaI Golden Gate Cloning; Km^R	**This Work**
**pNDMS494**	pNDGG012 with RmP110 pSymA nt 288,860 – 289,774 (GLND514/GLND515) via BsaI Golden Gate Cloning; Gm^R	**This Work**
**pNDMS495**	pNDGG013 with RmP110 pSymA nt 528,115 – 528,866 (GLND520/GLND521) via BsaI Golden Gate Cloning; Km^R	**This Work**
**pNDMS496**	pNDGG012 with RmP110 pSymA nt 539,707 – 540,936 (GLND522/GLND523) via BsaI Golden Gate Cloning; Gm^R	**This Work**

Table S2: Summary of Strains Used in Construction of Individual Deletions

Summary of Strains Used in Construction of Individual Deletions						
	First FRT Site Integration (Single Int.)			Second FRT Site Integration (Double Int.)		
Strain (Common Name)	Background	Plasmid Integrated	Resulting Strain	Background	Plasmid Integrated	Resulting Strain
**RmND868 (Deletion C2)**	RmP110	pNDMS493	RmND842	RmND842	pNDMS494	RmND861
**RmND869 (Deletion C3)**	RmP110	pNDMS495	RmND843	RmND843	pNDMS496	RmND862

Table S3: Primers for Inserts Used in Plasmid Construction

Primers for Inserts Used in Plasmid Construction					
**Vector**	**Primer**	**Forward**	**Primer**	**Reverse**	**Location**
**pNDMS493 (BsaI GG)**	**GLND512**	TTTGGTCTCAGGAGGGGGTCGGCGTTGTATTGAA	**GLND513**	TTTGGTCTCTAGCGCAAGGTGCGGTGTCTCTCC	RmP110 pSymA nt 286,239 – 286,590
**pNDMS494 (BsaI GG)**	**GLND514**	TTTGGTCTCAGGAGGCATCATTGAAGGAACCGTCG	**GLND515**	TTTGGTCTCTAGCGTCCAACGACCTCAGCGAATT	RmP110 pSymA nt 288,860 – 289,774
**pNDMS495 (BsaI GG)**	**GLND520**	TTTGGTCTCAGGAGTGAAATCATCGCCGGGTCG	**GLND521**	TTTGGTCTCTAGCGCTGGGCAGTTGAAGCGAATAC	RmP110 pSymA nt 528,115 – 528,866

**pNDMS496 (BsaI GG)**	**GLND522**	TTTGGTCTCAGGAGAGGGGATCAATGGGTTTGCC	**GLND523**	TTTGGTCTCTAGCGGCTCCCGCGTAATCCCTAG	RmP110 pSymA nt 539,707 – 540,936

Table S4: Primers Anchored in Genome Used for Verification of Plasmid Integration into Genome

**Primers Anchored in Genome Used for Verification of Plasmid Integration into Genome**				
**Plasmid**	**Primer**	**Forward**	**Primer**	**Reverse**
**pNDMS 493**	**GLND 516**	GGGAGAGGTGGCAGGAATTC	**GLND 517**	ACCATTTCGTACCCAAGCGC
**pNDMS 494**	**GLND 518**	GTCCTTCCGGTCCAATGTCG	**GLND 519**	ATCGACTCGTTGGCAGTGTT
**pNDMS 495**	**GLND 524**	TTTCCCCAAGCTCATCGACC	**GLND 525**	ATAGGCCAGCAACTCCAACC
**pNDMS 496**	**GLND 526**	ACCACTTCCTCCTGTTGCTG	**GLND 527**	CCACTACCTCTTCTTCGCCC

- Primers are based in genome bordering newly integrated plasmids. Only used for verification purposes.

Table S5: Primers Anchored in Vectors Used for Verification of Plasmid Integration into Genome

Primers Anchored in Vectors Used for Verification of Plasmid Integration into Genome				
**Vector**	**Primer**	**Forward**	**Primer**	**Reverse**
**pNDGG 012**	**GLND2 17**	TGTAGGTATCTCAGT TCGGT	**GLND2 18**	GCTGCTCCATAACAT CAAAC
**pNDGG 013**	**GLND2 19**	GCGTAATCTCTTGCT CTGAA	**GLND2 20**	CATCCAGTTTACTTT GCAGG

- Forward primers in vector backbones were paired with reverse primers anchored in genome, while reverse primers in vector backbones were paired with forward primers anchored in genome. Only used for verification purposes.

Table S6: Primer Pairings for Verification of Plasmid Integrations

Primer Pairings for Verification of Plasmid Integrations				
	**Left Region Verification Pairing**		**Right Region Verification Pairing**	
**Plasmid**	**Forward**	**Reverse**	**Forward**	**Reverse**
**pNDMS493**	GLND516	GLND220	GLND219	GLND517
**pNDMS494**	GLND518	GLND218	GLND217	GLND519
**pNDMS495**	GLND524	GLND220	GLND219	GLND525
**pNDMS496**	GLND526	GLND218	GLND217	GLND527

Table S7: Primer Pairings for Verification of the Excision of Flanked Region (Deletion Verification)

**Primer Pairings for Verification of the Excision of Flanked Region (Deletion Verification)**			
**Strain Name**	**Common Name**	**Forward**	**Reverse**
**RmND868**	Cluster 2 Deletion	GLND219	GLND519
**RmND869**	Cluster 3 Deletion	GLND219	GLND527

Supplementary figures

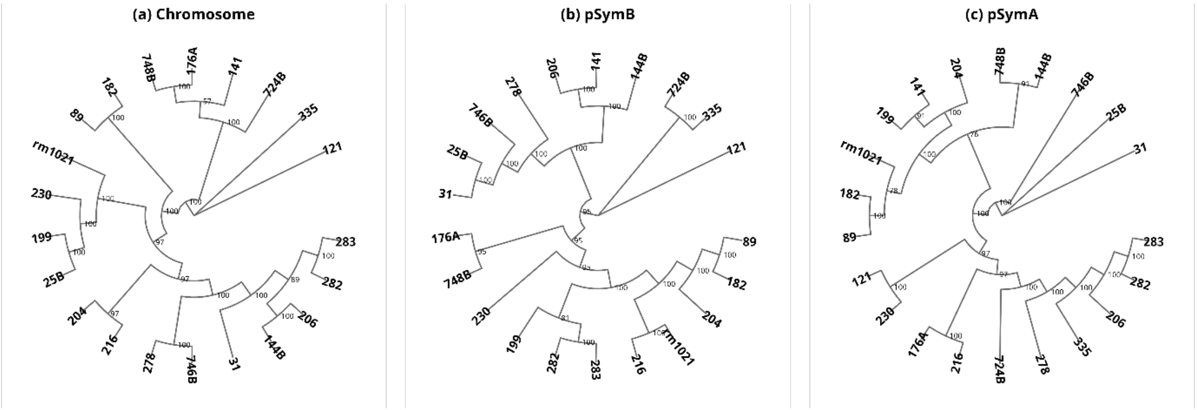

Figure S1.- Core phylogenies of the 20 *Sinorhizobium meliloti* wild isolates arranged by genetic replicon. Chromosome (a) pSymB (b) and pSymA (c). Bootstrap values are presented in nodes.

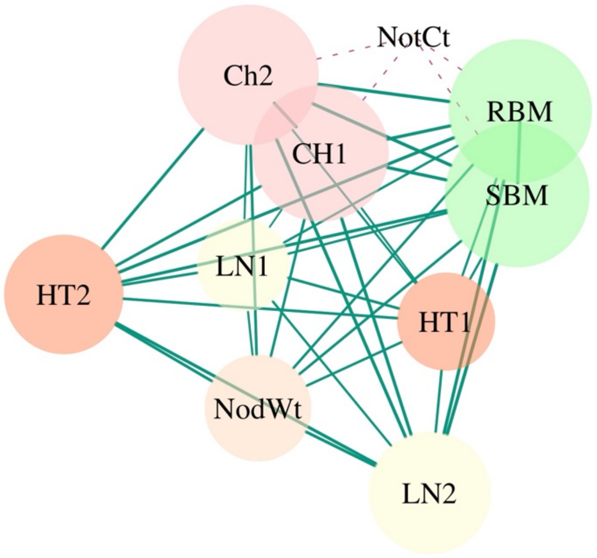

Figure S2: Trait-trait correlation network. Trait-Trait correlation (10 replicates per strain) network shows significant positive (green) and negative (pink) correlations between different traits/phenotypes; CH1 and CH2: early and late chlorophyll; HT1 and HT2: early and late height; LN1 and LN2: early and late leaf numbers; SBM: shoot biomass; RBM: root biomass; NodCt: nodule count; NodWt: nodule weight. Edges with a correlation coefficient of less than 0.3 are not shown, dotted lines represent |r| < 0.5, and edge width is proportional to the correlation coefficient.

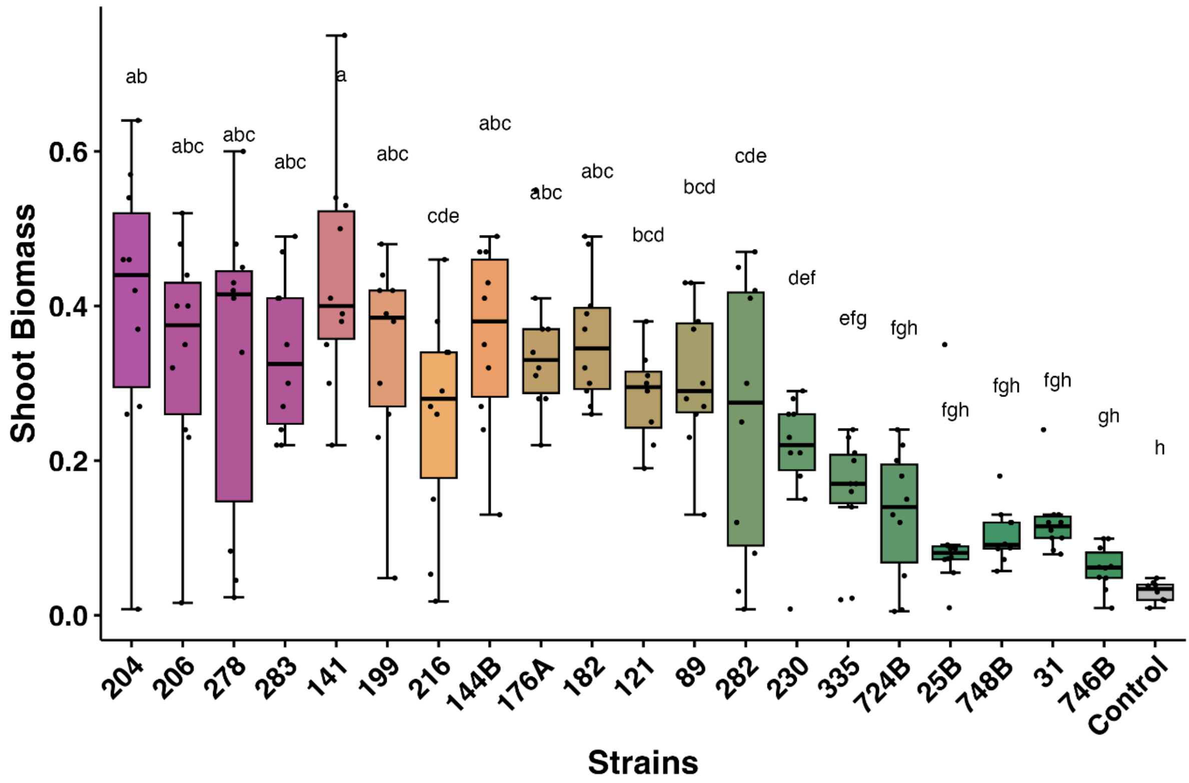

Figure S3: Partner quality of 20 *S. meliloti* strains. Boxplots present the distribution of plant biomass when *M. truncatula* host plants were inoculated with one of 20 strains (10 replicates each). Letter-based groupings of means were assigned by ANOVA followed by Duncan's test (alpha = 0.05).

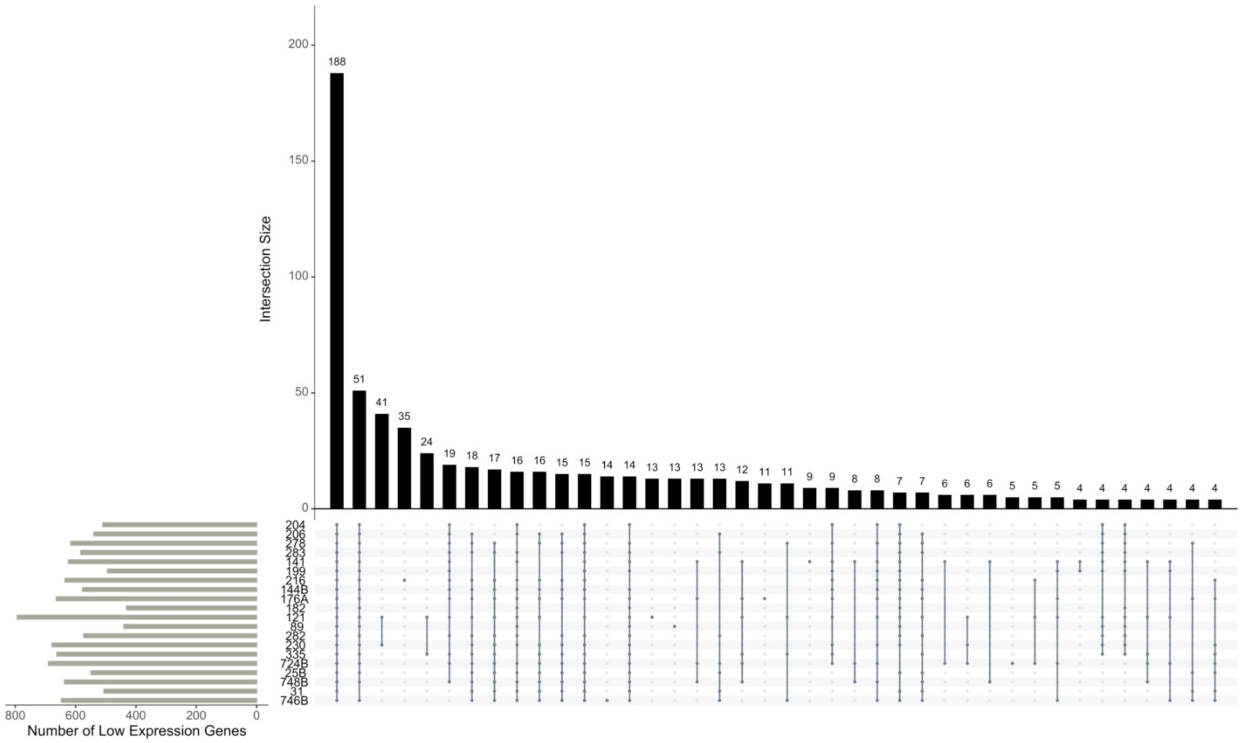

Figure S4: UpSet plot showing the distribution of low expression/absent genes among different strains. Each row corresponds to a specific strain, while columns represent the number of genes with very low/no expression in each strain, based on read alignment to the sm1021 reference. The horizontal bars show the number of genes absent in each strain, the vertical bars correspond to the number of intersecting/common genes among strains, and the dots represent the association between vertical bars and strains. The dots connected through lines show the overlap between those strains.

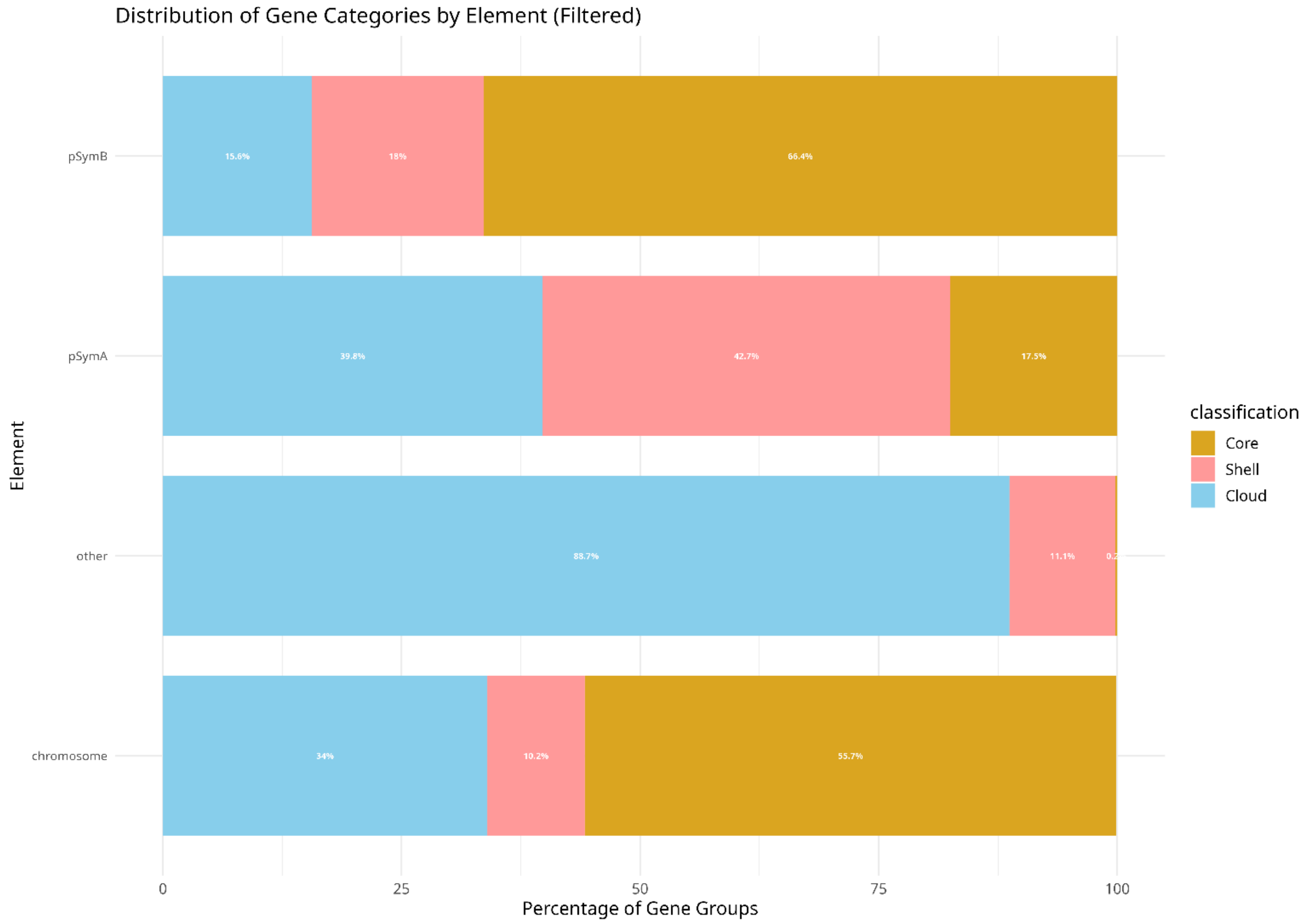

Figure S5.-Distribution of core, shell, and cloud gene groups across genomic elements. Proportion of core (gold) and accessory (shell in pink and cloud in blue) gene groups within four genomic elements. Core genes are found in 95–99% of strains, shell genes are found in 15–95%, while cloud genes are present in less than 15% of strains. Chromosome, pSymB, pSymA, and extra chromosomal undefined elements presented as “other”.

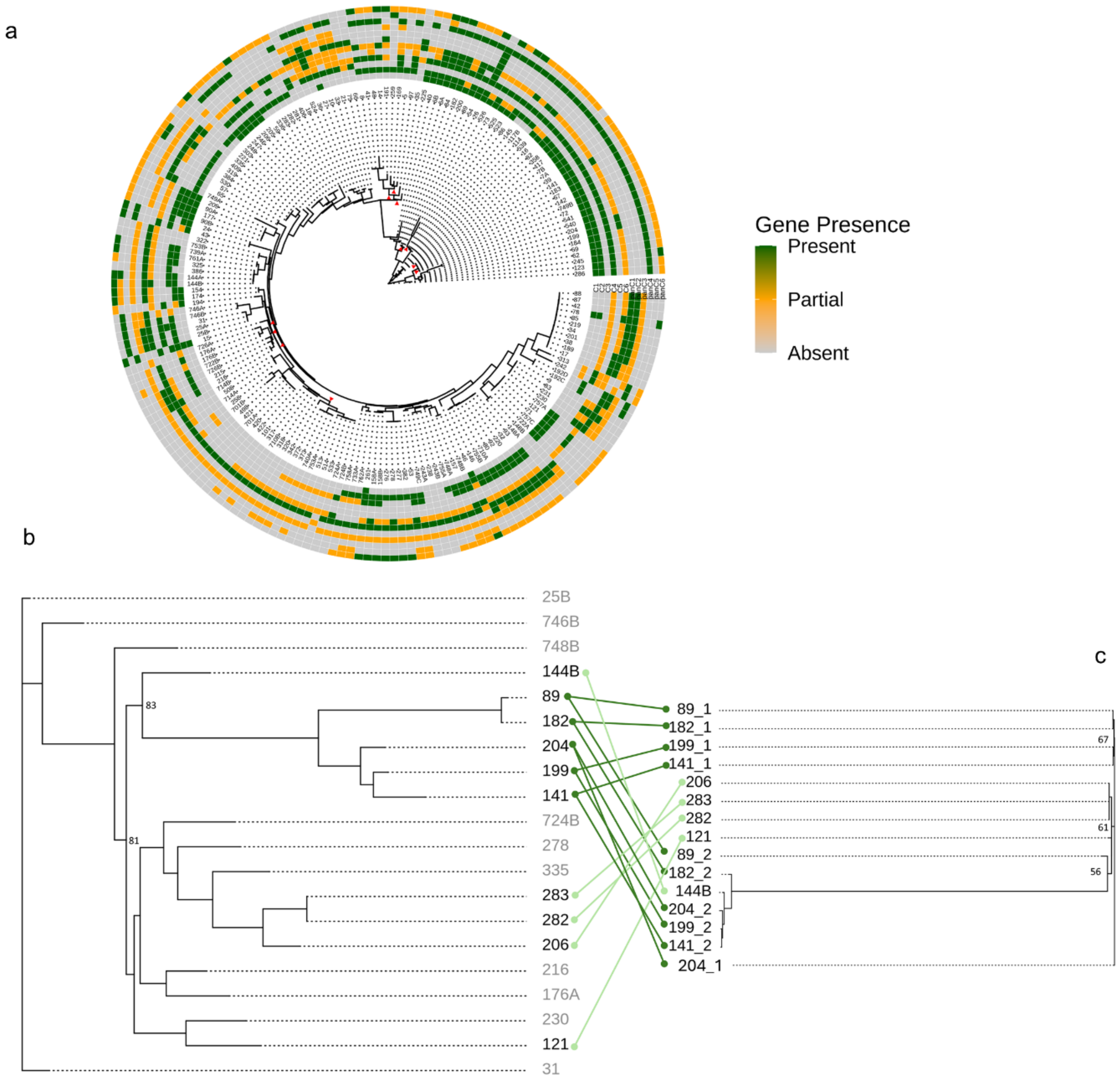

Figure S6: Phylogenies of the pSymA megaplasmid suggest independent mobilization of gene clusters. (A) The patterns of PAV of 12 clusters (C1-C6; panC1-panC6) across the core phylogeny of pSymA is displayed in the superset of 191 strains from Batstone *et al.* (30). Gray squares indicate cluster absence, while green squares indicate cluster presence, and yellow squares indicate partial presence, where at least one of the genes in the cluster is present. Red arrows point at the nodes with < 75% bootstrap support. (B) Core trees of pSymA of 20 focal strains indicating the presence (black) and absence (gray) pattern of cluster 2 (C) phylogeny derived from alignment of C2 gene sequences (in strains where present). Nodes with < 75% support are labeled. Bold green arrows indicate the topology connection between the two trees when two paralogs of cluster 2 are present. Faint green arrows indicate single copy topology connections between trees. Topology discordance is observed among trees indicating gains or losses of either one or two copies of C2.

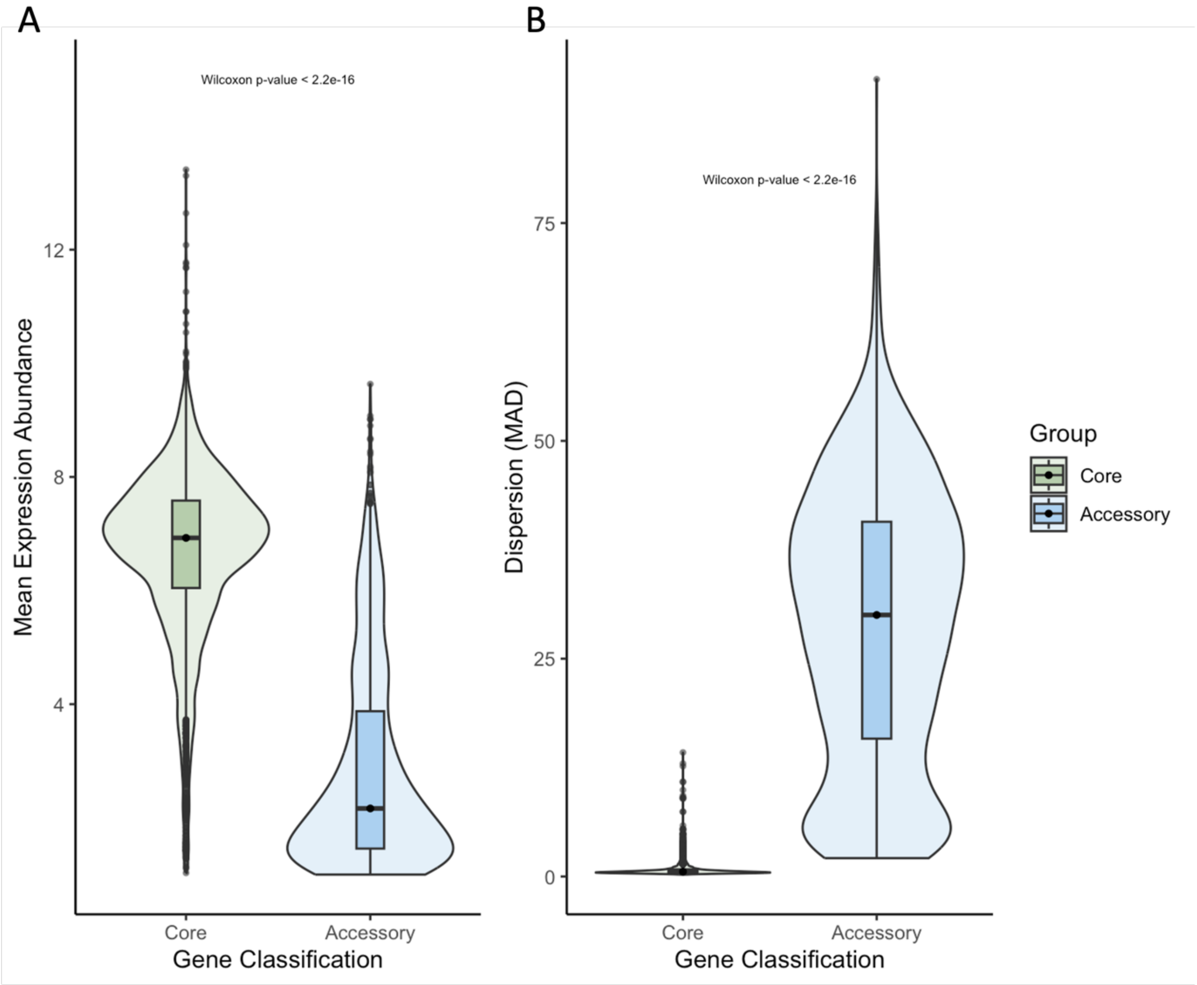

Figure S7. Comparison of gene expression abundance and dispersion between core and accessory genes. (A) Mean gene expression abundance was calculated as the mean log2 of the normalized read counts (counts per million (CPM)) across strains for which the gene was present in their genome. A two-sided Wilcoxon signed-rank test was performed on 4,926 core genes versus 2,073 accessory genes. (B) Gene dispersion was calculated as the mean absolute deviation (MAD). A two-sided Wilcoxon signed-rank test was performed to compare dispersion between core and accessory genes

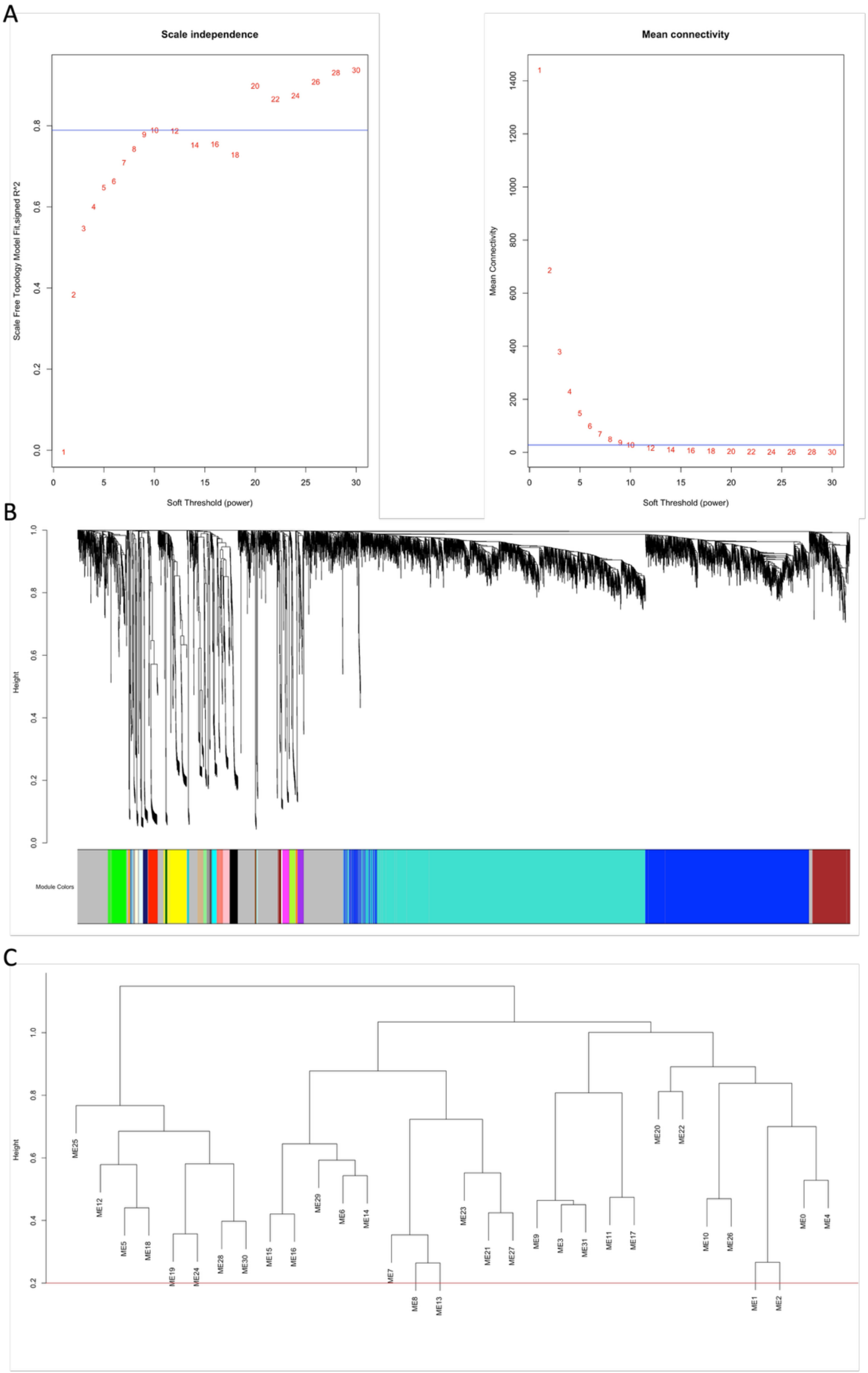

Figure S8: **Pangenome-based WGCNA of rhizobia genes**. The graph shows the relationship between soft-thresholding power (A) and network topology (B) with scale-free topology model fit (y-axis) and the soft-thresholding power (x-axis), as well as the mean connectivity (y-axis) in relation to the soft-thresholding power (x-axis). The lowest power (β=10) was selected where the scale-free topology fit index shows moderate mean connectivity. Genes

are grouped into distinct modules (M1–M31) based on topological overlap using clustering dendrograms, with each module assigned a unique color. Genes not assigned to any module are grouped in gray (M0). In (C), the dendrogram represents the clustering of module eigengenes, with each branch indicating a different module, revealing relationships and co-expression patterns among the gene networks by showing the similarity among module expression profiles.

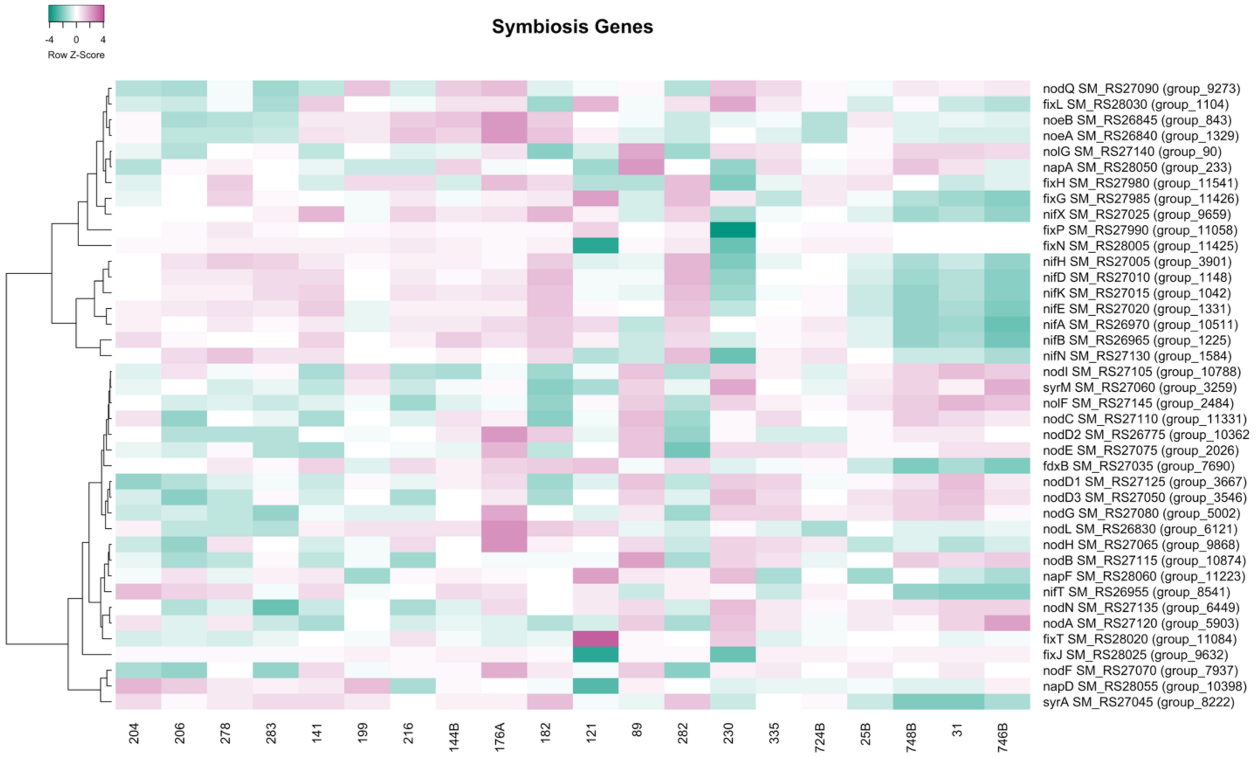

Figure S9: Gene expression of known symbiosis genes of rhizobia. Genes related to nodulation, nitrogen fixation, and symbiosis-related processes are selected (1, 42). Columns (strains) are sorted from high to low plant shoot biomass.

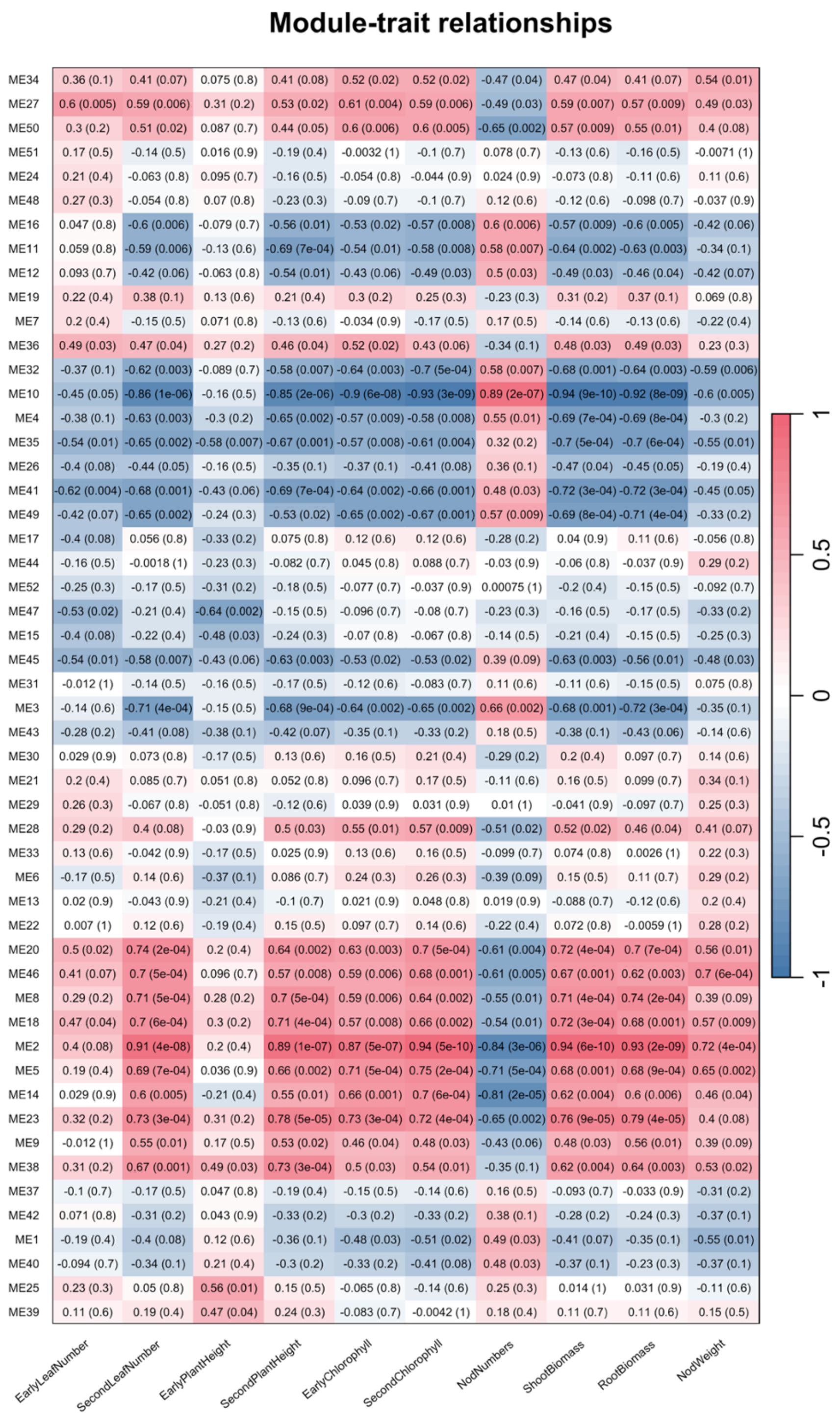

Figure S10: Module-Trait relationship heatmap of Medicago WGCNA network. (A) Each cell represents the correlation between module eigengene (row) and a specific trait or phenotype (column). The color gradient indicates the strength and direction of the correlation, with red representing positive correlations and blue representing negative correlations.

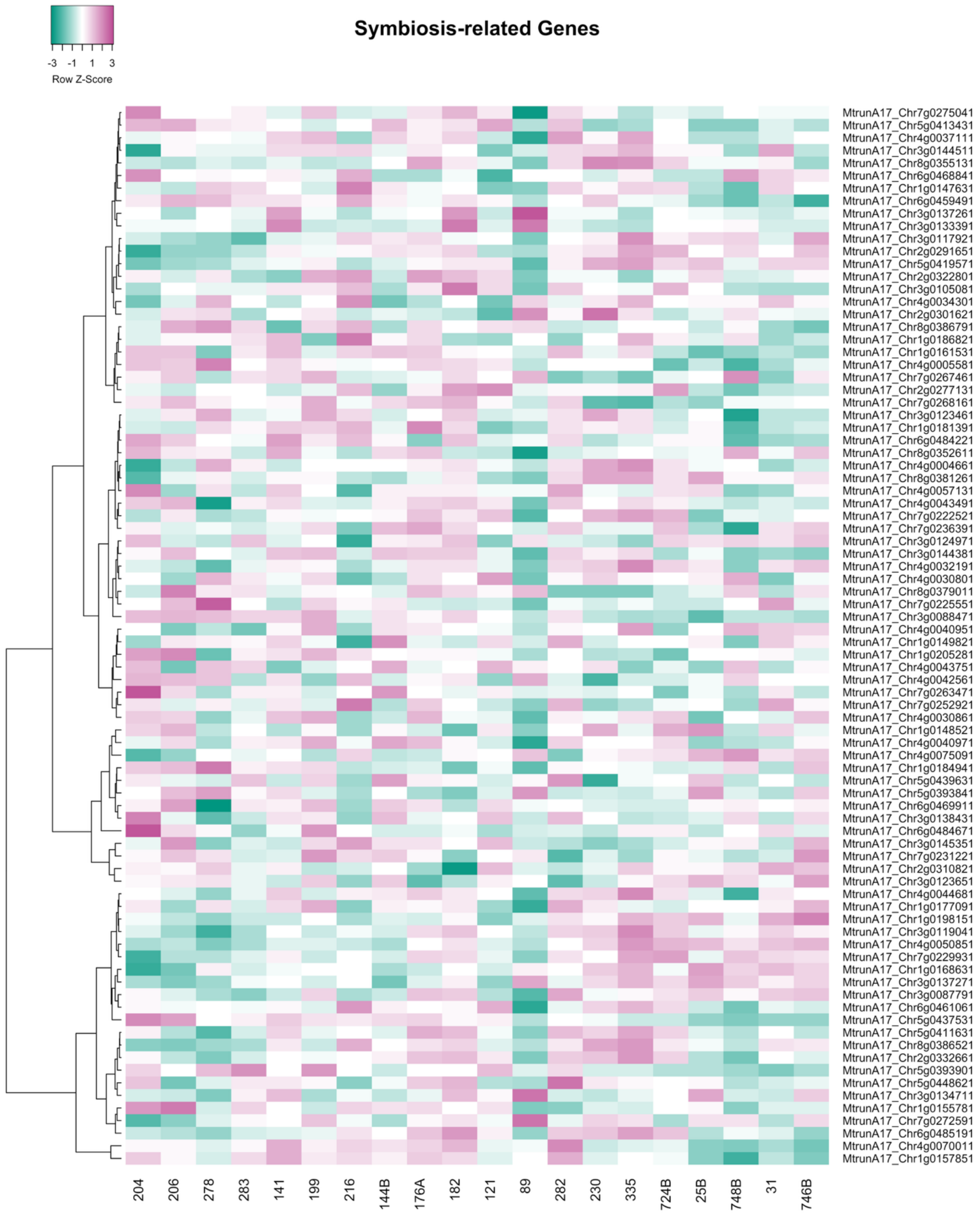

Figure S11: Symbiosis-related genes of *Medicago truncatula*. Genes were selected from a previous study (17) to show the expression profile of symbiosis-related genes. Columns are sorted from high to low plant shoot biomass.

Supplementary files

Dataset S1 (separate file). Strain means of 10 plant phenotypic traits, and ANOVA statistics used for Figure 1A and Figure S3.

Dataset S2 (separate file). Reads alignment information for 1021-based and pangenome mapping.

Dataset S3 (separate file). Presence-Absence Variation of 1021-based gene clusters (C1 – C6) and pangenome-based cluster (panC1 – panC6) significantly correlated with plant shoot biomass.

Dataset S4 (separate file). Summed to gene / gene cluster counts for all Medicago (49,928) and Ensifer pangenome (12,107) genes. Also includes the locus_tag IDs for strains 1021 and MABNR56, if in a gene cluster.

Dataset S5 (separate file). Raw counts of genes of panC1-panC6 clusters.

Dataset S6 (separate file). Module membership of *Sinorhizobium meliloti* pangenome gene groups in co-expression network.

Dataset S7 (separate file). COG enrichment terms of top modules of *Sinorhizobium meliloti* pangenome co-expression network.

Dataset S8 (separate file). Module membership of Medicago truncatula gene co-expression network.

Dataset S9 (separate file). Medicago and Ensifer WGCNA modules significantly correlated with each other and shoot biomass.

Dataset S10 (separate file). GO enrichment terms of top modules of *Medicago truncatula* co-expression network.

Dataset S11 (separate file). Module memberships and average logCPM expression of GWAS (Batstone et al.) universal candidates.

Dataset S12 (separate file). Preliminary genes identified from gene expression (presence-absence variation) correlation with shoot biomass in 1021-reference based analysis.

Dataset S13 (separate file). Module memberships of symbiosis-related genes in *Medicago truncatula* co-expression network.

References

1. B. A. Geddes *et al.*, Minimal gene set from Sinorhizobium (Ensifer) meliloti pSymA required for efficient symbiosis with Medicago. *Proc Natl Acad Sci U S A* **118**, e2018015118 , pmid = 2033384333 , publisher = National Academy of Sciences (2021).
2. M. J. Barnett *et al.*, Nucleotide sequence and predicted functions of the entire Sinorhizobium meliloti pSymA megaplasmid. *Proc Natl Acad Sci U S A* **98**, 9883-9888 (2001).
3. K. Jin *et al.*, Comparative Transcriptome Analysis of Agrobacterium tumefaciens Reveals the Molecular Basis for the Recalcitrant Genetic Transformation of Camellia sinensis L. *Biomolecules* **12**, 688 , pmid = 35625616 , publisher = MDPI (2022).
4. G. M. Ann, Attachment of Agrobacterium to plant surfaces. *Frontiers in Plant Science* **5**, 1-8 (2014).
5. A. L. Flores-Mireles, A. Eberhard, S. C. Winans, Agrobacterium tumefaciens can obtain sulphur from an opine that is synthesized by octopine synthase using S-methylmethionine as a substrate. *Mol Microbiol* **84**, 845-856 (2012).
6. P. J. Murphy *et al.*, Synthesis of an opine-like compound, a rhizopine, in alfalfa nodules is symbiotically regulated. *Proc Natl Acad Sci U S A* **85**, 9133-9137 (1988).
7. M. Sugawara *et al.*, Comparative genomics of the core and accessory genomes of 48 Sinorhizobium strains comprising five genospecies. *Genome Biol* **14**, R17 (2013).
8. J. Nogales, H. Blanca-Ordonez, J. Olivares, J. Sanjuan, Conjugal transfer of the Sinorhizobium meliloti 1021 symbiotic plasmid is governed through the concerted action of one- and two-component signal transduction regulators. *Environ Microbiol* **15**, 811-821 (2013).
9. D. Perez-Mendoza *et al.*, Identification of the rctA gene, which is required for repression of conjugative transfer of rhizobial symbiotic megaplasmids. *J Bacteriol* **187**, 7341-7350 (2005).
10. H. Blanca-Ordonez *et al.*, pSymA-dependent mobilization of the Sinorhizobium meliloti pSymB megaplasmid. *J Bacteriol* **192**, 6309-6312 (2010).
11. G. E. Wardell, M. F. Hynes, P. J. Young, E. Harrison, Why are rhizobial symbiosis genes mobile? *Philos Trans R Soc Lond B Biol Sci* **377**, 20200471 (2022).
12. P. Wangthaisong *et al.*, The Type IV Secretion System (T4SS) Mediates Symbiosis between Bradyrhizobium sp. SUTN9-2 and Legumes. *Appl Environ Microbiol* **89**, e0004023 (2023).
13. A. S. Fleischhacker, P. J. Kiley, Iron-containing transcription factors and their roles as sensors. *Curr Opin Chem Biol* **15**, 335-341 (2011).
14. L. Thony-Meyer, Biogenesis of respiratory cytochromes in bacteria. *Microbiol Mol Biol Rev* **61**, 337-376 (1997).
15. L. Trisolini *et al.*, FAD/NADH Dependent Oxidoreductases: From Different Amino Acid Sequences to Similar Protein Shapes for Playing an Ancient Function. *Journal of Clinical Medicine* **8** (2019).
16. N. Yan, Structural Biology of the Major Facilitator Superfamily Transporters. *Annual Review of Biophysics* **44**, 257-283 (2015).

17. S. Roy *et al.*, Celebrating 20 Years of Genetic Discoveries in Legume Nodulation and Symbiotic Nitrogen Fixation. *Plant Cell* **32**, 15-41 (2020).
18. E. L. Schnabel *et al.*, A Medicago truncatula Autoregulation of Nodulation Mutant Transcriptome Analysis Reveals Disruption of the SUNN Pathway Causes Constitutive Expression Changes in Some Genes, but Overall Response to Rhizobia Resembles Wild-Type, Including Induction of TML1 and TML2. *Curr Issues Mol Biol* **45**, 4612-4631 (2023).
19. Y. Pecrix *et al.*, Whole-genome landscape of Medicago truncatula symbiotic genes. *Nat Plants* **4**, 1017-1025 (2018).
20. E. W. Sayers *et al.*, Database resources of the national center for biotechnology information. *Nucleic Acids Res* **50**, D20-D26 (2022).
21. F. Galibert *et al.*, The composite genome of the legume symbiont Sinorhizobium meliloti. *Science* **293**, 668-672 (2001).
22. R. Patro, G. Duggal, M. I. Love, R. A. Irizarry, C. Kingsford, Salmon provides fast and bias-aware quantification of transcript expression. *Nat Methods* **14**, 417-419 (2017).
23. R. C. Team (2022) R: A Language and Environment for Statistical Computing.
24. C. Soneson, M. I. Love, M. D. Robinson, Differential analyses for RNA-seq: transcript-level estimates improve gene-level inferences. *F1000Res* **4**, 1521 (2015).
25. D. R. a. A. O. Mark, A scaling normalization method for differential expression analysis of RNA-seq data. *Genome Biology* **11**, 1-9 (2010).
26. F. Almeida-Silva, T. M. Venancio, BioNERO: an all-in-one R/Bioconductor package for comprehensive and easy biological network reconstruction. *Funct Integr Genomics* **22**, 131-136 (2022).
27. M. D. Robinson, D. J. McCarthy, G. K. Smyth, edgeR: a Bioconductor package for differential expression analysis of digital gene expression data. *Bioinformatics* **26**, 139-140 (2010).
28. C. W. Law, M. Alhamdoosh, S. Su, G. K. Smyth, M. E. Ritchie, RNA-seq analysis is easy as 1-2-3 with limma, Glimma and edgeR. *F1000Research* **5**, 1408 (2016).
29. J. R. Conway, A. Lex, N. Gehlenborg, UpSetR: an R package for the visualization of intersecting sets and their properties. *Bioinformatics* **33**, 2938-2940 (2017).
30. C. Camacho *et al.*, BLAST+: architecture and applications. *BMC Bioinformatics* **10**, 421 (2009).
31. A. Rhoads, K. F. Au, PacBio Sequencing and Its Applications. *Genomics Proteomics Bioinformatics* **13**, 278-289 (2015).
32. R. R. Wick *et al.*, Trycycler: consensus long-read assemblies for bacterial genomes. *Genome Biol* **22**, 266 (2021).
33. A. J. Collins, R. J. Whitaker, CRISPR Comparison Toolkit: Rapid Identification, Visualization, and Analysis of CRISPR Array Diversity. *CRISPR J* **6**, 386-400 (2023).
34. G. Tonkin-Hill *et al.*, Producing polished prokaryotic pangenomes with the Panaroo pipeline. *Genome Biol* **21**, 180 (2020).
35. E. Caudal *et al.*, Pan-transcriptome reveals a large accessory genome contribution to gene expression variation in yeast. *Nat Genet* **56**, 1278-1287 (2024).
36. B. Milunovic, G. C. diCenzo, R. A. Morton, T. M. Finan, Cell growth inhibition upon deletion of four toxin-antitoxin loci from the megaplasmids of Sinorhizobium meliloti. *J Bacteriol* **196**, 811-824 (2014).

37. T. M. Finan, B. Kunkel, G. F. De Vos, E. R. Signer, Second symbiotic megaplasmid in Rhizobium meliloti carrying exopolysaccharide and thiamine synthesis genes. *J Bacteriol* **167**, 66-72 (1986).
38. B. A. Geddes *et al.*, BEVA2.0: modular assembly of golden gate-compatible vectors with expanded utility for genetic engineering. *Can J Microbiol* **71**, 1-13 (2025).
39. P. Langfelder, S. Horvath, WGCNA: an R package for weighted correlation network analysis. *BMC Bioinformatics* **9**, 559 (2008).
40. Z. C. Yuan, R. Zaheer, T. M. Finan, Regulation and properties of PstSCAB, a high-affinity, high-velocity phosphate transport system of Sinorhizobium meliloti. *J Bacteriol* **188**, 1089-1102 (2006).
41. Y. Zhang, T. Aono, P. Poole, T. M. Finan, NAD(P)+-malic enzyme mutants of Sinorhizobium sp. strain NGR234, but not Azorhizobium caulinodans ORS571, maintain symbiotic N2 fixation capabilities. *Appl Environ Microbiol* **78**, 2803-2812 (2012).
42. F. Ampe, E. Kiss, F. Sabourdy, J. Batut, Transcriptome analysis of Sinorhizobium meliloti during symbiosis. *Genome Biol* **4**, R15 (2003).
